# Human milk oligosaccharides regulate human macrophage polarization and activation in response to *Staphylococcus aureus*

**DOI:** 10.3389/fimmu.2024.1379042

**Published:** 2024-06-06

**Authors:** Stine Dam Jepsen, Astrid Lund, Martin Matwiejuk, Lars Andresen, Kristine Rothaus Christensen, Søren Skov

**Affiliations:** ^1^ dsm-firmenich, Hørsholm, Denmark; ^2^ Immunology, Section for Preclinical Disease Biology, Department of Veterinary and Animal Sciences, University of Copenhagen, Frederiksberg, Denmark

**Keywords:** human milk oligosaccharides, myeloid activation, immunology, 6′-sialyllactose, 2′-fucosyllactose, lacto-N-neotetraose, *Staphylococcus aureus*

## Abstract

Human milk oligosaccharides (HMOs) are present in high numbers in milk of lactating women. They are beneficial to gut health and the habitant microbiota, but less is known about their effect on cells from the immune system. In this study, we investigated the direct effect of three structurally different HMOs on human derived macrophages before challenge with *Staphylococcus aureus* (*S. aureus*). The study demonstrates that individual HMO structures potently affect the activation, differentiation and development of monocyte-derived macrophages in response to *S. aureus*. 6´-Sialyllactose (6’SL) had the most pronounced effect on the immune response against *S. aureus*, as illustrated by altered expression of macrophage surface markers, pointing towards an activated M1-like macrophage-phenotype. Similarly, 6’SL increased production of the pro-inflammatory cytokines TNF-α, IL-6, IL-8, IFN-γ and IL-1β, when exposing cells to 6’SL in combination with *S. aureus* compared with *S. aureus* alone. Interestingly, macrophages treated with 6’SL exhibited an altered proliferation profile and increased the production of the classic M1 transcription factor NF-κB. The HMOs also enhanced macrophage phagocytosis and uptake of *S. aureus*. Importantly, the different HMOs did not notably affect macrophage activation and differentiation without *S. aureus* exposure. Together, these findings show that HMOs can potently augment the immune response against *S. aureus*, without causing inflammatory activation in the absence of *S. aureus*, suggesting that HMOs assist the immune system in targeting important pathogens during early infancy.

## Graphical Abstract

Created with BioRender.com.

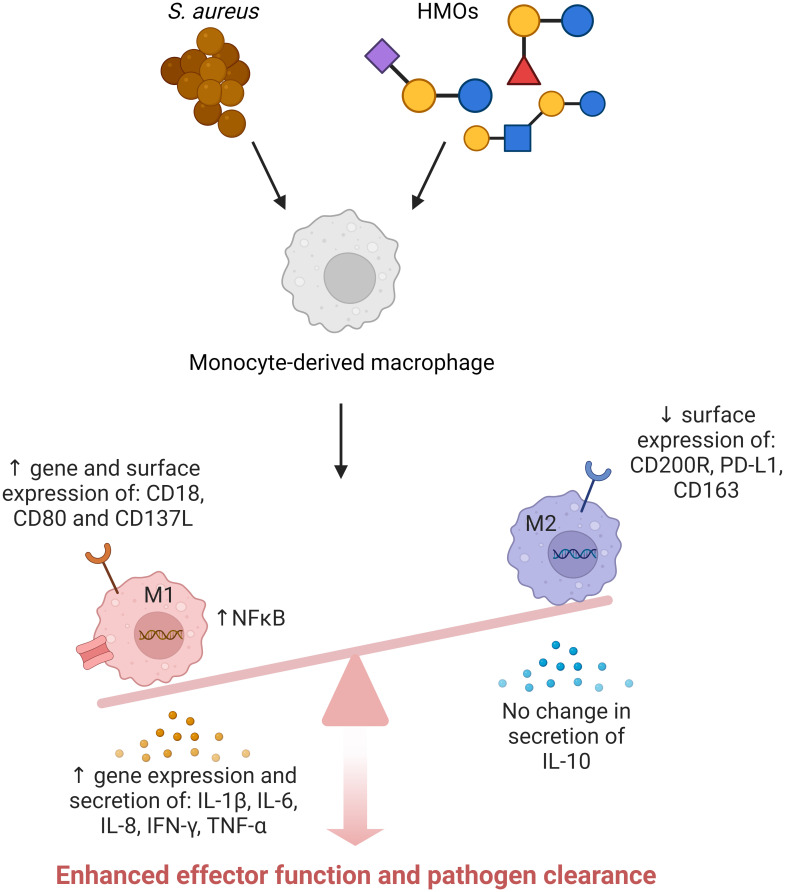

## Introduction

Human breast milk, the first and primary nutrition source for infants, is rich in nutrients and bioactive compounds that support development and growth (1, 2). Human milk oligosaccharides (HMOs) are complex sugar molecules, and the third most prevalent solid component in human breast milk. More than 200 different HMOs have been identified; however, only 10-15 structures make up the majority of the biomass (3). HMOs can be divided into three groups depending on their structural composition: 1) Non-fucosylated neutral core HMOs, made up of a neutral core backbone linked to one or more N-acetylglucosamine units; 2) Fucosylated neutral core HMOs with one or more fucose units; or 3) Sialylated HMOs with one or more sialylated units. HMOs are beneficial to microbiota and gut homeostasis (4–6), but have also shown to inhibit pathogen binding by functioning as decoy receptors (7, 8), and they can indirectly shape the intestinal immune system by producing immunomodulatory fermentation products, such as short chain fatty acids (9–14). HMOs have also been shown to affect the gut ecosystem and immune system in the absence of microbiota, as evaluated in germ-free mice (15). HMOs are able to regulate the human intestinal cell line HT-29 (16) and Zhang et al. revealed that a small fraction of HMOs with a high degree of polymerization increased the production of IL-1β, IL-6, IL-2, IL-10 and TNF-α in the murine macrophage cell line RAW264.7, and increased the expression of MAPKs and intracellular ROS (17). Likewise, Xiao et al. showed that a mixture of HMOs affected human monocyte derived dendritic cells, by increasing maturation, cytokine production, migration markers and anti-inflammatory properties in T cells (18). Recently, Boll et al. demonstrated that HMOs have structure-dependent functions and that HMOs belonging to the group of non-fucosylated neutral core and the group of fucosylated neutral core have beneficial effects on the epithelial cells, whereas HMOs belonging to the sialylated HMO-group have immunomodulatory functions in dendritic cells and macrophages (19). Lastly, the addition of HMOs to infant formula was demonstrated to reduce inflammatory cytokine production to resemble cytokine profiles of breastfed infants (20). Together, this strongly shows that HMOs can regulate cells of the immune system. Still, a thorough investigation of individual HMO structures and their effect on macrophage polarization is needed to fully understand the direct effect of individual HMOs.

Monocytes are blood-borne immune cells and when monocytes extravasate to tissues, they mature into macrophages. Monocytes and macrophages can differentiate in one of two directions: M1-like, which causes a potent immune response that is beneficial when combatting pathogens, and M2-like, that takes part in anti-inflammatory tissue remodeling (21). In the gut, the balance between the macrophage phenotypes is fine-tuned, and imbalances can cause persistent infection or chronic inflammation (22). The balance between M1 and M2-like states are dynamic, and macrophages are often in a state between the two extremes (23). Recently, changes in intracellular metabolism have been shown to be involved in this dynamic regulation of monocytes and macrophages (24). Several immune-relevant receptors found on macrophages are known for their glycan-binding properties, including C-type lectins, galectins and siglecs (25–27). Noll et al. described 2’FL binding to DC-SIGN (28), and have also previously described HMO binding to galectins (29). A similar finding was done by Hassan et al. demonstrating that 3′-Sialyllactose (3’SL) binds galectin-1 and galectin-3 (30). Finally, siglecs are sialic acid binding lectins, relevant in immune cell signaling and endocytosis (26, 31) and have been shown to be able to bind sialylated HMOs (32).

*Staphylococcus aureus* (*S. aureus*) is a commensal bacterium, colonizing 30-40% of the human population (33, 34). *S. aureus* is mainly present on the skin, in the nasopharyngeal tract and in the gut microbiota (35). The bacterium is often asymptomatic, but is characterized as an opportunistic pathogen and can cause damage ranging from mild and superficial infections to more severe disease such as bacteremia (36). Breastfed infants are at high risk of getting *S. aureus* from their mother’s skin, if the mothers are asymptomatic carriers (37, 38). *S. aureus* in the gut have been hypothesized to help develop the intestinal immune system (39, 40), but the intestines can also be an entry point for systemic dissemination. *S. aureus* can switch from being a harmless colonizer to an infectious pathogen, depending on the interplay between the gut, immune cells, microbiota, as well as gut integrity (41). *S. aureus* also have a potential to evade the immune system and develop antibiotic resistance (42, 43), and therefore relevant alternatives to antibiotics are of major interest in the treatment of *S. aureus* infections.

Monocytes and macrophages are highly relevant players in combatting *S. aureus* infections (35, 44). An abundance of M2-macrophages is associated with persistent infection and biofilm formation (45). Therefore, it is potentially relevant to enhance the M1-like macrophages to defeat infection (46, 47). Once the infection has been overcome, it is important that the homeostatic balance is reestablished to avoid excess damage.

The aim of this study was to investigate the direct effect of individual HMO structures on macrophage activation and polarization. *S. aureus* was chosen for macrophage activation in order to get a physiological relevant response, compared to artificial TLR ligand activation. The study shows that HMOs, especially 6´-Sialyllactose (6’SL), are able to significantly enhance effector functions of macrophages exposed to *S. aureus*. The results include increased expression of pro-inflammatory genes and surface molecules, enhanced cytokine responses, increased cell proliferation and nuclear factor kappa-light-chain-enhancer of activated B cells (NF-κB) induction and increased uptake of *S. aureus*. No noticeable macrophage activation was apparent in the absence of *S. aureus* challenge, demonstrating the safety of HMO-imposed immune regulation. This highlights the ability of HMOs to enhance specific effector functions in macrophages when encountering pathogens, which may be important for development of gut homeostasis and battle against infectious diseases.

## Materials and methods

### Cells and culture conditions

#### Primary cells, purification

Primary monocytes were isolated from buffy coats of healthy human volunteers obtained from the National Hospital (Copenhagen, Denmark). Monocytes were isolated using RosetteSep Human Monocyte Enrichment Cocktail (StemCell, 15068), followed by density centrifugation in Histopaque (Sigma, 10771) according to the manufacturer’s instructions. Red blood cells were removed by incubation in red blood cell-lysis buffer (eBioscience, 00-4333-57) for 10 minutes, and subsequently the cells were washed in PBS (Sigma, D8537) supplemented with 2% FBS (Sigma, F9665). Isolated monocytes were either set up for experiment or frozen in 90% FBS (Sigma, F9665) and 10% DMSO (Sigma, D2438), until further use.

#### Culture conditions

Primary monocytes and cell lines were cultivated in RPMI 1640 media (Sigma, R5886) supplemented with 10% FBS (Sigma, F9665), 2 mM L-glutamine (Sigma, G7513), and 2 mM penicillin/streptomycin (Sigma, P4333), henceforth referred to as standard 1640 RPMI media. The human monocytic (pre-macrophage) cell line U937 was purchased from Sigma (Sigma, 85011440) and the THP1–NF-kB–GFP reporter cells were kindly provided by Dr. Peter Steinberger (Institute of Immunology, Medical University of Vienna, Vienna, Austria) (48).

### Human milk oligosaccharides

**Figure 1:**
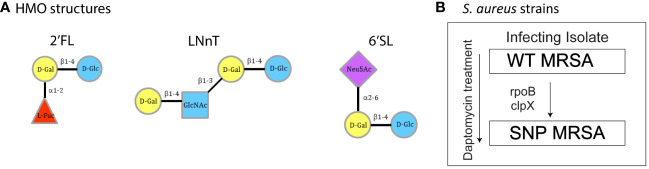
Overview of HMO structures and *S. aureus* strains. **(A)** Structural composition of the three individual HMO structures used in this experiment: 2’FL, LNnT and 6’SL. **(B)** Clinical *S. aureus* strains WT MRSA and SNP MRSA. The arrow indicates acquired SNPs and decreased daptomycin susceptibility. WT MRSA is the infecting isolate and SNP MRSA have decreased daptomycin susceptibility. Figure B modified from Mellergaard et al. (43, 49). Un, Untreated; 2’FL, 2′-Fucosyllactose; LNnT, Lacto-N-neotetraose; 6’SL, 6’-Sialyllactose; D-Gal, D-Galactose; D-Glc, D-Glucose; L-Fuc, L-Fucose; Glc-NAc, N-Acetylglucosamine; Neu5AC, N-Acetylneuraminic acid.

Chemically synthesized HMOs and HMOs produced by fermentation were prepared in house by Glycom A/S (Hørsholm, Denmark). Their structures were verified by 1H and 13C-NMR. Endotoxin measurements on HMOs produced by fermentation were performed with the Limulus Amebocyte Lysate Kinetic-QCL™ from Lonza (US License No. 1775, catalog No.:50-650U). HMO structures used in this study are depicted in **Figure 1A**.

#### HMO-preparation for cell culture

Endotoxin-free HMOs were prepared in standard RPMI 1640 media at 37°C to a concentration of 20mM, passaged through a 0,2 µm sterile filter (Satorius, 16534 —GUK) and used immediately after preparation.

### Preparation of UV-inactivated *S. aureus* strains

The clinically derived methicillin resistant *S. aureus* (MRSA) WT MRSA (SADR1/A9781) and SNP MRSA (SADR2/A9788) strains were previously described (original names in brackets) (49–51). Culture and UV-inactivation of *S. aureus* were performed as previously described (49). In brief, overnight *S. aureus* colonies were inoculated in trypsin soy broth to OD_600_ ~ 0.03 and grown in Erlenmeyer flasks to early stationary phase (OD600 = 5-6). Afterwards, the bacteria were washed and resuspended in PBS to OD600 = 1 (~10^9^ *S. aureus*/ml). *S. aureus* solutions were transferred to petri dishes and subjected to pulsed UV-radiation of 10.000μJ/cm^2^ for 120 seconds (monochromatic wavelength of 254nm; CL-1000 crosslinker; UVP, Cambridge, United Kingdom) for UV-inactivation. Bacterial death was verified by plating and incubating on TSA plates at 37°C overnight (**Supplementary Figure 1**).

### Primary macrophages: cell treatment and experimental set up

Primary monocytes were isolated from buffy coats, as described above. The isolated monocytes were seeded at 3 x 10^5^ cells/ml and cultured in standard RPMI 1640 media, supplemented with 20 mM of HMO and 40 ng/mL M-CSF (Peprotech, 300-25). On day three, the cells received half new medium supplemented with the concentration of HMOs and M-CSF doubled. On day 6 all medium was discarded and replaced with fresh standard RPMI 1640 media containing 20 mM of HMO. The cells were then treated with 12,5 x 10^6^ *S. aureus*/ml cell suspension and the monocyte-derived macrophages were further activated with *S. aureus* strains for two days. On day 8 the cells were taken for analysis.

### Total RNA purification and transcriptomic profiling

Total RNA purification of primary monocytes was completed on day 8, after cultivation, as described above. Cell media was removed, the cells were lysed in RLT buffer (Qiagen, 79216) and stored at -80°C until purification. Total RNA purification was performed on a QIAcube Connect Instrument using the RNeasy Mini QIAcube Kit (Qiagen, 74116) with on-column DNase treatment, according to the manufacturer’s instructions. Purity and concentration were measured on a Denovix DS11 spectrophotometer. RNA quality was assessed on a bioanalyzer. An A260/A280 ratio above 1.95 and a RIN score above 7 was required for transcriptomic analysis. Hereafter RNA was stored at -20°C until further analysis. Transcriptomic profiling was done using Clariom™ D Assay (ThermoFischer Scientific, 902922) and performed by Center for Genomic Medicine, Copenhagen University Hospital. Data analysis was done using Transcriptome Analysis Console 4.0.2 (ThemoFischer Scientific). Mean probe signal intensity on each chip was used to normalize for loading differences between chips. Fold change values were reported by the program.

### Flow cytometry

#### Counting

Adherent macrophages were counted by removing the supernatant and loosening the cells. Two different detachment methods were evaluated: A) Trypsination using trypsin/EDTA solution (Sigma, T4049) for 10 min at 37°C and resuspending the cells thoroughly: or B) Incubation of cells in citric saline for 15 min at 37°C followed by cell scraping using a cell lifter (Fischerbrand, 11577692). Afterwards, the cells were transferred to a 96-well plate and counted on a MacsQuant 16 Flow Cytometer.

#### Surface marker staining and analysis

Adherent cells were detached in citric saline followed by cell scraping using a cell lifter. For surface staining, cells were washed twice in cold FACS buffer containing PBS + 2% FBS, incubated with FcR Blocking Reagent (Miltenyi Biotec, 130-059-901) for 10 min on ice, and then washed and stained with fluorescent antibodies for 15 min on ice. Additionally, two washes were performed, and the cells were resuspended and analyzed in FACS buffer. Cells were analyzed with a MacsQuant 16 Flow Cytometer and data analysis was done using Flowlogic 8.6 Software and MACSQuantify™ 2.13.3 software. For every sample, technical duplicates were analyzed. Gating was carried out on viable cells (**Supplementary Figure 2**) and the results are presented as percent or mean fluorescence intensity average for technical duplicates (MFI). Isotype controls were included in the study and are presented in the supplementary section (**Supplementary Figure 3**). Specific antibodies (**Supplementary Table 1**) and isotypes controls (**Supplementary Table 2**) are listed in the supplementary section.

### Cytokine measurements

Levels of secreted cytokines were measured in harvested cell culture supernatants. The analysis was performed using chemiluminescence-based assays from Meso Scale Discovery (MSD, Gaithersburg, MD, USA). The studies included two different assays from the V-PLEX platform: V-PLEX Human IL-8 Kit (K151RAD) singleplex and V-PLEX Viral Panel 2 Human Kit (K15346D). Assay set up, execution and analysis were done according to the manufacturer’s instructions. The analysis was performed using QuickPlex Q 120 instrument (MSD) and DISCOVRY WORKBENCH^®^ 4.0 software.

### NF-κB reporter assay

Investigation of the NF-κB-pathway was done using THP1-NF-κB-GFP reporter cells, kindly provided by Dr. Peter Steinberger (48). The reporter cells were seeded at a concentration of 3 x 10^5^ cells/ml in standard RPMI media supplemented with HMO and incubated overnight at 37°C, 5% CO_2_. The next day *S. aureus* bacteria were added to the cells at a concentration of ~50 x 10^6^ bacteria/ml cell suspension, and again incubated overnight at 37°C, 5% CO_2_. The GFP-signal was evaluated by flow cytometry the following day. The cells were harvested, transferred to a 96-well plate, and washed twice in PBS + 2% FBS, before analysis by flow cytometry.

### Cell proliferation

To evaluate cell-proliferation, monocytes were stained with Carboxyfluorescein Succinimidyl Ester (CFSE, Molecular Probes, C34554) prior to seeding as previously described (49, 52). Shortly, this was done by dissolving cells in PBS + 5% FBS to a concentration of 5 x 10^6^ cells/ml. The CFSE stock was diluted according to the manufacturer’s manual and added 1:1 to the cell suspension for final concentration of 5 μM. The cells were incubated dark for 5 min, while rotating at room temperature. Afterwards the cells were washed twice in PBS + 5% FBS and resuspended in standard 1640 RPMI media, supplemented with HMOs and M-CSF and set up as described above.

### Brightfield microscopy

On day 8, prior to analysis, the cells were inspected in the microscope and photographed in the petri dish at 10x optics. Photos were taken using the ECHO Rebel Microscope.

### Incucyte photos

Monocytic derived macrophages were cultured in the presence of 20 mM HMOs and 40 ng/mL M-CSF as earlier described and photographed on day 7. The photos of the cells were taken using Incucyte^®^, SX1 Live-Cell Analysis System.

### Fluorescent labeling of UV-killed *S. aureus*

UV inactivated *S. aureus* strains were labelled with AF647 conjugated succinimidylester (SE-AF647; Molecular Probes, A-20006), as described in (49). Briefly, bacteria were resuspended in the original volume of sodium bicarbonate buffer (pH 8.5), SE-AF647 was added at a concentration of 10 ng/μL and bacteria were incubated at 4°C for 1 h with vigorous agitation. Stained bacteria were washed and resuspended to original volume in PBS and stored at -20°C until use. The staining was confirmed on the flow cytometer (**Supplementary Figure 4**). Data was acquired with an Accuri C6 instrument using Accuri C6 software and analyzed in Flowlogic 8.6 Software.

### Uptake of fluorescent labelled and UV-killed *S. aureus* in U937 cells

U937 cells were seeded at a concentration of 3 x 10^5^ cells/ml in standard 1640 RPMI media supplemented with 20 mM HMO and incubated overnight at 37°C, 5% CO_2_. The next day, UV-killed SE-AF647 labelled *S. aureus* bacteria were added to the cells at a concentration of ~50 x 10^6^ bacteria/ml cell suspension and incubated overnight at 37°C, 5% CO_2_. The following day, the uptake of UV-killed SE-AF647-labelled *S. aureus* bacteria was evaluated. Cells were transferred to a 96-well plate, and washed twice in PBS + 2% FBS, before analyzing them by flow cytometry.

### Phagocytosis of *S. aureus* bioparticles in primary macrophages

Primary monocytes were cultured as previously stated, supplemented with 20 mM HMOs and 40 ng/mL M-CSF (Peprotech, 300-25) for a total of 8 days. On day 8, 0,1μg *S. aureus* bioparticles (pHrodo^®^ Red *S. aureus* Bioparticles^®^ for Incucyte^®^, 4619) were added in 50µl/well (30.000 cells/well) in a 96-well plate. The plate was incubating for 15 minutes at room temperature to let the particles descend. The phagocytic uptake was analyzed by Incucyte^®^, SX1 Live-Cell Analysis System.

### Figures

The graphical abstract was created in BioRender.com. Publication license, including confirmation of publication and licensing rights are available under agreement number is *RM26AK4PC3*.

### Statistical analysis

Data preparation and statistical analysis was performed using GraphPad Prism, version 9.3.1 (GraphPad Software). Statistical analysis was performed as stated in the figure legends, and the level of statistical significance was determined by a p-value of less than 0.05 and is presented as follows: *p < 0.05, **p < 0.01, ***p < 0.001 and ****p < 0.0001.

## Results

In this study, two previously described human infectious strains of *S. aureus* were used (49, 50). WT MRSA is the infecting isolate, whereas SNP MRSA is isolated from the same patient with bacteremia, after initiation of daptomycin treatment (**Figure 1B**). SNP MRSA has acquired two SNP mutations, in rpoB and clpP, known to be associated with antibiotic-resistance and immune evasion (49, 50). Here, we investigated the effect of individual HMO structures on macrophages exposed to an infecting *S. aureus* strain (WT MRSA). In addition to this primary focus, we explored the effect of individual HMO structures on the antibiotic-resistant SNP MRSA strain, since antibiotic resistance is an increasing problem affecting economy and global health (53). Results from challenge with the SNP MRSA strain are included throughout this article, but are specifically addressed at the end of the results section.

### HMOs alter macrophage relevant genes towards an M1 phenotype in response to *S. aureus*

**Figure 2:**
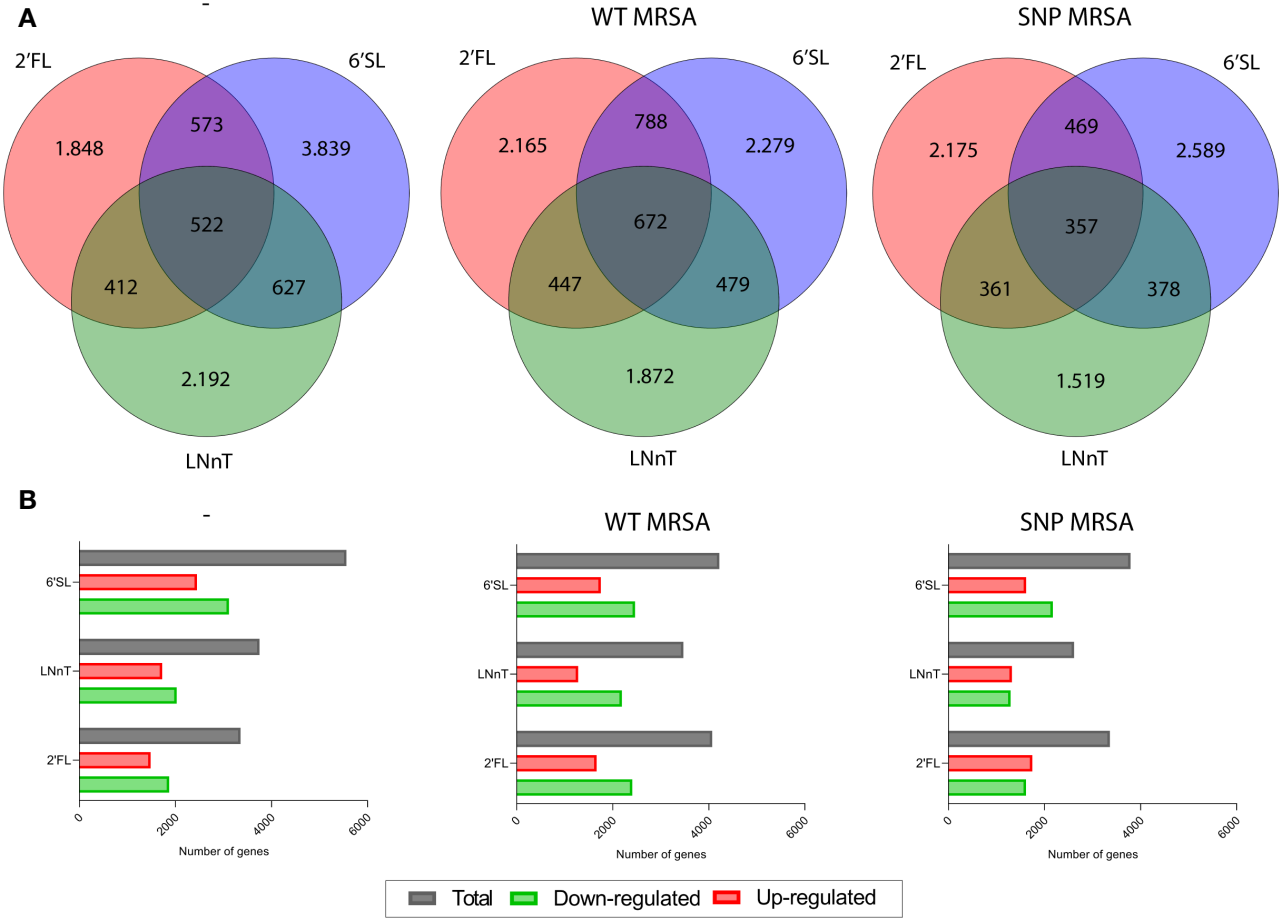
HMOs alter gene expression of several genes in monocyte-derived macrophages. Monocytes were treated with HMOs followed by treatment with WT MRSA or SNP MRSA. On day 8, total RNA was isolated, followed by transcriptomic profiling. Data is presented as **(A)** Venn diagrams and **(B)** bar graphs with data points showing number of differentially expressed genes in untreated macrophages and macrophages treated with WT MRSA and SNP MRSA from one experiment. Data is presented as number of genes with a fold change of < -2 or > 2 relative to untreated sample. 2’FL, 2′-Fucosyllactose; LNnT, Lacto-N-neotetraose; 6’SL, 6’-Sialyllactose.

To investigate the effect of individual HMO structures on macrophages in response to *S. aureus*, transcriptomic profiling was performed on human blood monocyte-derived macrophages. Monocytes were isolated from healthy donors and treated with three different HMOs; 2’FL, LNnT or 6’SL (**Figure 1A**), as well as M-CSF, followed by treatment with *S. aureus*. On day 8, transcriptomic analysis was performed on total RNA, which showed that all three HMOs changed gene regulation of a high number of genes (**Figure 2**). The highest number of genes affected is seen in 6’SL-stimulated macrophages challenged with WT MRSA as depicted in **Figures 2A, B**. We observed an overlap in gene-regulation between the HMOs, but clearly the different structures also hold very specific regulatory potential (**Figure 2A**).

### HMOs change surface expression of macrophage-relevant proteins to mimic an M1 phenotype in response to *S. aureus*

To further explore the effect of HMOs on macrophages in response to *S. aureus*, the gene expression of several phenotypic macrophage-related surface molecules was investigated. From transcriptomic profiling, we found that all three HMO structures altered several macrophage-related surface proteins in response to *S. aureus*. Specifically, macrophages exposed to 6’SL had increased transcripts for the costimulatory activation marker CD137L (**Supplementary Figure 5A**), costimulatory molecule CD80 (**Supplementary Figure 5B**) and adhesion molecule CD18 (**Supplementary Figure 5C**). Concurrently, a decrease in the M2-markers CD200R and CD163 (**Supplementary Figures 5D, F**) was observed. The different responses are linked to the presence of *S. aureus* and are not notably affected by macrophage exposure to 6’SL alone. This indicates a boosted immune response against *S. aureus*.

**Figure 3:**
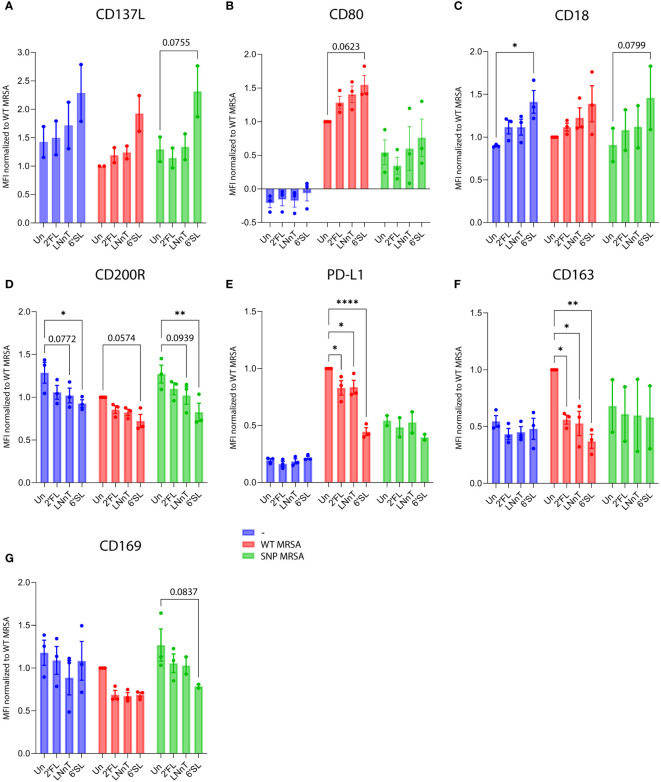
HMOs drive macrophage surface proteins towards a M1-characteristic phenotype in response to *S. aureus*. Monocytes were treated with HMOs followed by treatment with WT MRSA and SNP MRSA and analyzed by flow cytometry on day 8. Data is presented as bar graphs with data points showing cell surface expression of **(A)** CD137L, from two independent experiments and **(B)** CD80, **(C)** CD18, **(D)** CD200R, **(E)** PD-L1, **(F)** CD163 and **(G)** CD169 from three independent experiments. Data is presented as MFI values ± SEM, normalized to WT MRSA untreated. Statistical analysis was performed by 2-way ANOVA with Dunnett’s multiple comparison test and presented relative to untreated control. MFI, Mean Fluorescent Intensity; Un, Untreated; 2’FL, 2′-Fucosyllactose; LNnT, Lacto-N-neotetraose; 6’SL, 6’-Sialyllactose. *p < 0.05, **p < 0.01 and ****p < 0.0001.

To examine if the altered gene expression correlated with subsequent surface expression, human blood-derived monocytes were exposed to combinations of M-CSF, HMOs and *S. aureus* by flow cytometry. Data is gated on viable cells (**Supplementary Figure 2**). Isotype controls were included in all surface marker studies but showed no unspecific binding (**Supplementary Figure 3**). Moreover, cells were CD14 positive, indicating myeloid origin (**Supplementary Figure 6**). Surface molecule expression results confirmed that 6’SL is the most potent of the three HMO structures evaluated. Following *S. aureus* challenge 6’SL increased cell surface expression of the activating molecule CD137L (**Figure 3A**; **Supplementary Figure 7A**), the co-stimulatory molecule CD80 (**Figure 3B**; **Supplementary Figure 7B**) and adhesion molecule CD18 (**Figure 3C**; **Supplementary Figure 7C**), and at the same time decreased the anti-inflammatory and myeloid suppressive surface molecules CD200R, PD-L1 and CD163 in combination with *S. aureus* (**Figures 3D–F**; **Supplementary Figures 7D–F**). Interestingly, CD169 (Siglec-1) also decreased in the presence of HMOs and *S. aureus* (**Figure 3G**; **Supplementary Figure 7G**). Expression of additional classical myeloid cell surface molecules were analyzed but showed no difference (**Supplementary Figure 8**).

These findings support the results from the gene array analysis and illustrates that most proteins regulated by HMOs on a transcriptional level are subsequently expressed on the cell surface. This is true for most of the displayed surface molecules. As an exception, *S. aureus*-induced gene expression of PD-L1 was potentiated by 6’SL and 2’FL, but did not translate into a higher surface expression. Contrarily, *S. aureus*-induced surface expression of PD-L1 was lower upon addition of HMOs (**Figure 3E**; **Supplementary Figure 7E**).

### Treatment with HMOs increases secretion of pro-inflammatory cytokines from macrophages in response to *S. aureus*

Cytokine-genes were also potently regulated by HMOs (**Supplementary Figure 9**). Again, 6’SL was the most potent of the three tested HMOs, although 2´FL and LNnT also impacted cytokine gene transcription (**Supplementary Figures 9A, B, D, E, G**). The data show that macrophages treated with 6’SL before *S. aureus* challenge stimulate an increased production of genes encoding the pro-inflammatory cytokines: TNF-α, IL-6, IL-8, IFN-γ and IL-1β (**Supplementary Figures 9A, D, E, F, G**), and simultaneously decreased gene expression of the anti-inflammatory cytokine IL-10 (**Supplementary Figure 9B**). As with the expression of surface proteins, the HMO effect on the expression of pro-inflammatory cytokines is dependent on the presence of *S. aureus* bacteria for the majority of the tested cytokines.

**Figure 4:**
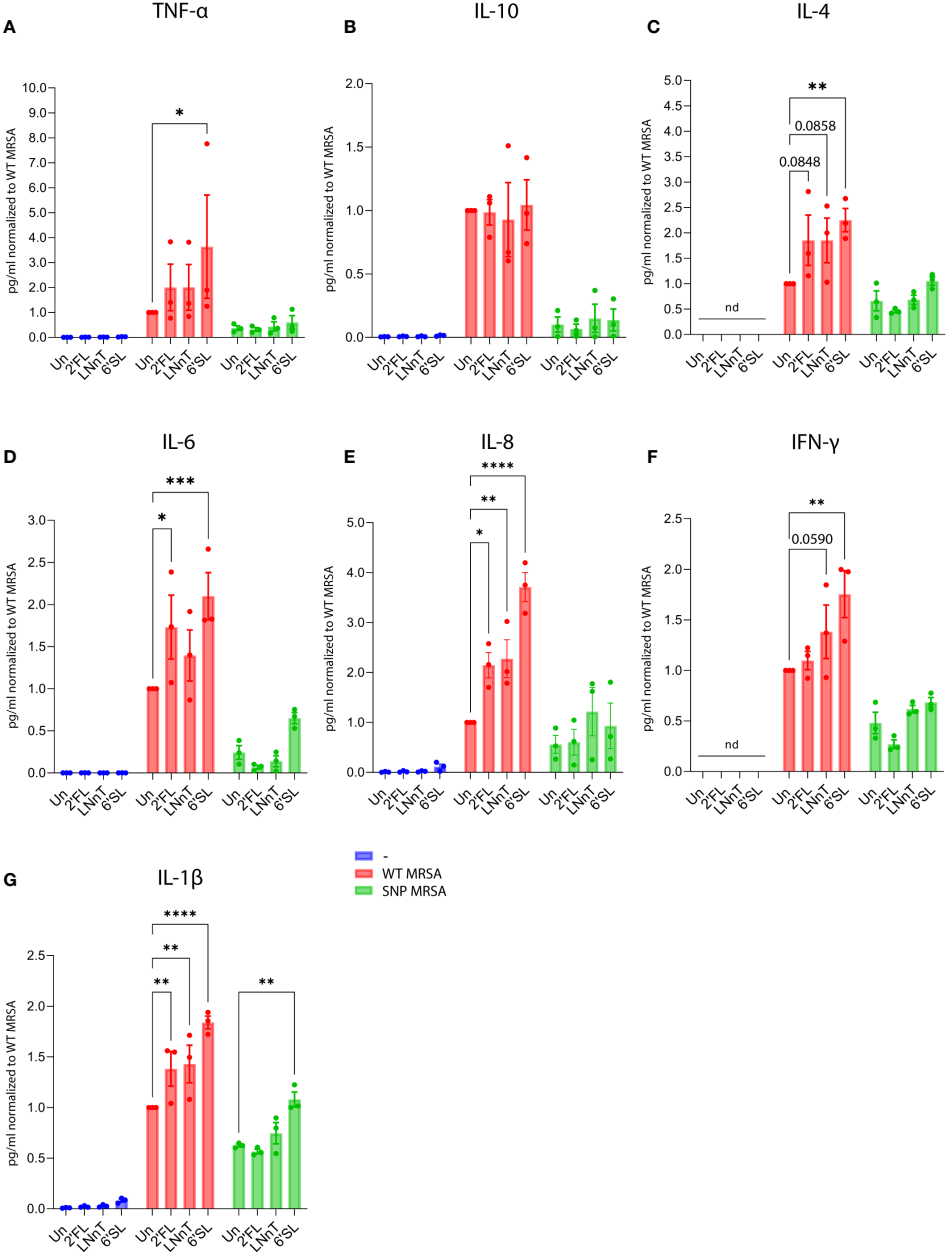
Treatment with HMOs increases secretion of pro-inflammatory cytokines from macrophages in response to *S. aureus*. Monocytes were treated with HMOs followed by treatment with WT MRSA and SNP MRSA. On day 8, cytokine levels were measured in culture supernatants by Mesoscale analysis. Bar graphs with data points show concentration of secreted cytokines normalized to WT MRSA untreated sample. Data represents three independent experiments, showing **(A)** TNF-α, **(B)** IL-10, **(C)** IL-4, **(D)** IL-6, **(E)** IL-8, **(F)** IFN-γ and **(G)** IL-1β. Concentration of cytokines were adjusted to the number of cells and presented as mean ± SEM, normalized to WT MRSA untreated. Statistical analysis was performed by 2-way ANOVA with Dunnett’s multiple comparison test. ND, Not detected; Un, Untreated; 2’FL, 2′-Fucosyllactose; LNnT, Lacto-N-neotetraose; 6’SL, 6’-Sialyllactose. *p < 0.05, **p < 0.01, ***p < 0.001 and ****p < 0.0001.

In order to further investigate HMO-regulated secretion of cytokines, monocyte-derived macrophages were stimulated as described above to collect supernatants for cytokine measurements. The cytokine levels were measured by mesoscale multiplex analysis, and cell numbers were evaluated by flow cytometer-mediated counting. To ensure that the macrophage detachment method did not affect the read-out, two different methods were tested: Trypsination and citric saline (54) combined with cell scraping. As shown in **Supplementary Figure 10**, there was no difference between the methods, and the citric saline combined with cell scraping method was applied to enable subsequent flow cytometry analysis of the cells. In parallel with the observation from the transcriptomic profiling, HMO treatment (in particular 6’SL) followed by *S. aureus* challenge increased the pro-inflammatory cytokines TNF-α, IL-6, IL-8, IFN-γ and IL-1β (**Figures 4A, D–G**). No change in the regulation of the anti-inflammatory cytokine IL-10 was observed (**Figure 4B**), whereas IL-4 increased relative to the untreated control (**Figure 4C**). In summary, these observations are aligned with the results from the gene array and cell surface analysis, indicating that HMOs can boost the inflammatory response against the *S. aureus* pathogen without affecting unexposed macrophages.

### HMOs increase activation of the NF-κB-pathway in response to *S. aureus* in THP1 reporter cell line

**Figure 5:**
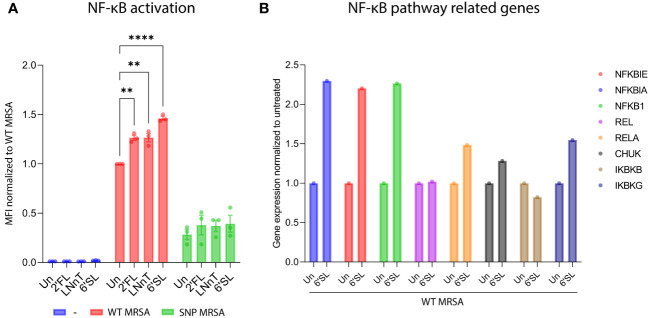
HMOs increases activation of the NF-κB-pathway as a response to *S. aureus* in a THP1 reporter cell line and NF-κB-related genes in macrophages. **(A)** NF-κB-THP1 reporter cell line was treated with HMOs followed by treatment with WT MRSA and SNP MRSA and analyzed by flow cytometry. Data is presented as bar graph with data points showing GFP-signal as a measure for NF-κB activation. Data represents three independent experiments and is presented as GFP-MFI values ± SEM normalized to untreated WT MRSA sample. The statistical analysis was performed by 2-way ANOVA with Dunnett’s multiple comparison test and presented relative to untreated control. **(B)** Monocytes were treated with 6’SL followed by treatment with WT MRSA. On day 8, total RNA was isolated, and genes relevant to the NF-κB-pathway were investigated by transcriptomic profiling. Data is presented as gene expression relative to untreated control and represents one experiment. MFI, Mean Fluorescent Intensity; NF-κB, Nuclear factor kappa-light-chain-enhancer of activated B cells; Un, Untreated; 2’FL, 2′-Fucosyllactose; LNnT, Lacto-N-neotetraose; 6’SL, 6’-Sialyllactose. **p < 0.01 and ****p < 0.0001.

Next, we investigated the potential of HMOs to activate the NF-κB-pathway. The NF-κB-pathway is a common pathway activated during infection and inflammation (55). We used a monocytic THP1-reporter cell line that expresses GFP in response to NF-κB activation, and it is therefore comprehensive to monitor myeloid cell activation by flow cytometry. As shown in **Figure 5A**, all tested HMOs increased the inflammatory NF-κB-response against *S. aureus*. Furthermore, the HMOs did not induce NF-κB-activity in macrophages without exposure to bacteria, highlighting that the effect is linked to the presence of pathogens (**Figure 5A**). Since HMO-mediated NF-κB activation was only observed together with *S. aureus* exposure, and as NF-κB can drive pro-inflammatory responses in macrophages (55), this could likely be a causative factor in the regulation of macrophages. Evaluation of data from microarray assay revealed that multiple genes relevant in the NF-κB-pathway were upregulated in macrophages treated with 6’SL and challenged with WT MRSA (**Figure 5B**). More specifically, there was an upregulation of the genes NFKBIE, NFKBIA, NFKB1, RELA, CHUK and IKBKG with 6’SL compared to untreated control. REL and IKBKB showed no difference in response to 6’SL. These upregulations were not seen in absence of the WT MRSA bacteria nor with the other HMOs (data not shown), pointing to a specific effect of 6’SL against pathogens.

### Chemically synthesized and fermentation-produced HMOs show same myeloid activation profile

**Figure 6:**
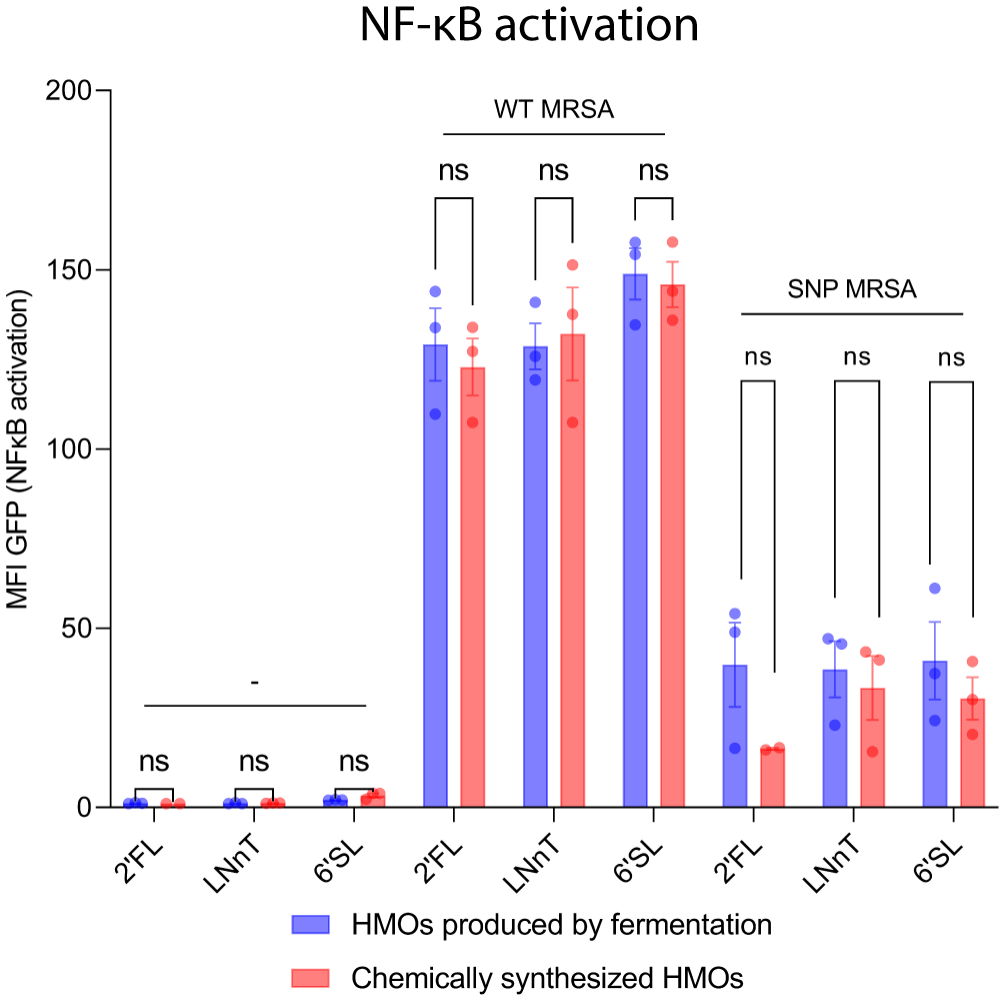
Chemically synthesized HMOs and HMOs produced by fermentation show same myeloid activation profile. NF-κB-THP1 reporter cells were treated with HMOs followed by treatment with WT MRSA and SNP MRSA and analyzed by flow cytometry. Data is presented as a bar graph with data points showing the GFP-signal ± SEM as a measure for NF-κB activation and represents three individual experiments. Statistical analysis was performed by 2-way ANOVA with Sidak´s multiple comparison test and presented as difference between fermented-produced- and chemically synthesized HMOs. Note that some of the data points included in this figure is identical to data in **Figure 5**. MFI, Mean Fluorescent Intensity; NF-κB, Nuclear factor kappa-light-chain-enhancer of activated B cells; Un, Untreated; HMOs, Human Milk Oligosaccharides; 2’FL, 2′-Fucosyllactose; LNnT, Lacto-N-neotetraose; 6’SL, 6’-Sialyllactose; ns, Not significant.

Most manufactured HMOs are produced by a fermentation process (56). To rule out that byproducts from the manufacturing process could impact or stimulate the immune cells, we did a comparison between HMOs produced by fermentation and HMOs produced chemically. Both fermented and chemically produced HMOs caused NF-κB activation, and there was no difference in activation between fermented and chemically produced HMOs (**Figure 6**). Moreover, an endotoxin analysis was performed on the fermented HMOs to rule out presence of endotoxins. The endotoxin test could not detect endotoxins above the assay threshold level (**Table 1**), clearly suggesting that the effect of HMOs is not caused by endotoxins. The studies in this article were carried out with the commercially available fermented HMOs.

**Table 1:** HMOs used in this study.

HMO	Synthetic origin	Batch	Purity	Endotoxin level
**2’ FL**	**Chemically**	**L06112K**	**99.9%**	**n/a**
**2’FL**	**Fermentation**	**20156002**	**98.2%**	**< 0.00025 EU/mg**
**LNnT**	**Chemically**	**L01032K**	**93.6%**	**n/a**
**LNnT**	**Fermentation**	**20135001**	**99.4%**	**< 0.00025 EU/mg**
**6’SL**	**Chemically**	**3CGH1169**	**86.8%**	**n/a**
**6’SL**	**Fermentation**	**19487101**	**98.8%**	**< 0.00025 EU/mg**

The purity, endotoxin level and origin of three different HMO structures used in this study. Further details of the compounds can be found in the related patents (57–59).

### HMOs alter the proliferation and morphology of macrophages

Microscopic inspection showed that HMOs affected growth activation patterns. Therefore, proliferation of macrophages was evaluated by CFSE-labelling and culturing with combinations of M-CSF, HMOs and *S. aureus*. On day 8, the macrophages were analyzed by flow cytometry. As seen in **Supplementary Figures 11A, B**, cells receiving HMOs, most significant 6’SL, proliferated more compared to untreated controls, shown as a decrease in CFSE-MFI value, compared to untreated control (**Supplementary Figure 11B**). Microscopic evaluation further corroborated that 6’SL treatment affected macrophage differentiation and morphology by making them more adherent and elongated (**Supplementary Figure 12**). Together this indicates an altered activation profile for macrophages exposed to 6’SL compared with the untreated control.

### HMOs increase *S. aureus* uptake by monocytes and phagocytosis in primary macrophages

**Figure 7:**
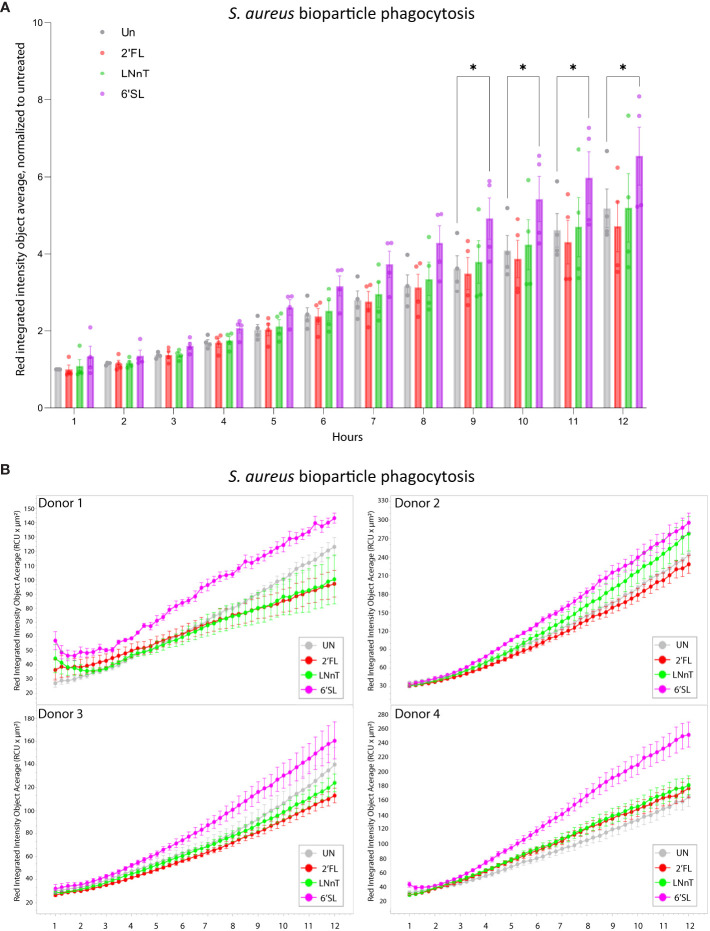
Treatment with HMOs increases phagocytosis of *S. aureus* bioparticles in primary macrophages. Primary macrophages were cultured for 8 days in the presence of 20 mM HMOs. On day 8 *S. aureus* bioparticles were added to the cells and the uptake was analyzed for 12 hours. Data is presented as **(A)** bar graph with data points showing red integrated intensity object average, normalized to untreated control at T-1h ± SEM from four individual experiments and **(B)** line graph representing the red integrated intensity object average over time for four donors (presented separately) ± SEM of three technical replicates. Statistical analysis was performed by 2-way ANOVA with Dunnett’s multiple comparison test and presented relative to untreated sample. Un, Untreated; 2’FL, 2′-Fucosyllactose; LNnT, Lacto-N-neotetraose; 6’SL, 6’-Sialyllactose. *p < 0.05.

Having established that the HMOs can push macrophages towards an M1-like phenotype, the ability of HMOs to affect bacterial uptake was evaluated in U937 monocytes, using fluorescent-labelled *S. aureus* (**Supplementary Figure 4**). Each HMO increased the uptake of *S. aureus*, suggesting that the phagocytic potential is augmented by HMOs (**Supplementary Figures 13A, B**). We further analyzed the phagocytic uptake by primary macrophages cultured with HMOs (**Figures 7A, B**; **Supplementary Figure 13C**). Here 6’SL significantly enhanced the phagocytosis of the *S. aureus* bioparticles compared to the 2’FL, LNnT and the untreated control. Together, this indicates that HMOs, and in particular 6’SL, have the potential to increase both the inflammatory and phagocytic response against a bacterial infection, in this case *S. aureus*.

### HMOs enhance macrophage response upon exposure to *S. aureus* with mutations linked to antibiotic resistance

We further investigated the effect of HMOs on macrophages against a more antibiotic-resistant *S. aureus* strain. The SNP MRSA strain has clpP and rpoB SNP-mutations, related to increased antibiotic-resistance. The HMOs, especially 6’SL, increased the pro-inflammatory phenotype against the more antibiotic-resistant SNP MRSA, but to a lower extent than that observed for WT MRSA. This include increased gene expression of the adhesion molecule CD18 (**Supplementary Figure 5C**), and decreased gene expression of CD200R, CD163 and D169 (**Supplementary Figures 5D, F, G**). HMOs also increased gene expression of pro-inflammatory cytokine genes, including TNF, IL-6, IL-8 and IL-1β (**Supplementary Figures 9A, D, E, G**), and decreased the gene expression of IL-10 (**Supplementary Figure 9B**). When evaluating the effect of HMOs on surface expression of several proteins, 6’SL showed similar effect when co-cultured with SNP MRSA as when co-cultured with WT MRSA. This includes an increase in activating molecule CD137L and adhesion molecule CD18 (**Figures 3A, C**), and a decrease in the anti-inflammatory surface molecules CD200R and PD-L1 (**Figures 3D, E**), as well as CD169 (**Figure 3G**). Regulation of the co-stimulatory molecule CD80 and the M2-marker CD163 was not affected by SNP MRSA exposure, suggesting that the more resistant SNP MRSA has a potential to evade the immune system, as previously shown (43). However, HMOs can indeed activate macrophages against the resistant *S. aureus* strain. In agreement with this, the response of pro-inflammatory cytokines IL-6, IL-8 and IL-1β (**Figures 4D, E, G**) was enhanced by co-cultivating SNP MRSA with 6’SL. The macrophage proliferation was also higher in response to SNP MRSA, when co-culturing with HMOs (**Supplementary Figure 11**). In addition, a slight increase in SNP MRSA uptake was observed in U937 monocytes pretreated with 2’FL (**Supplementary Figures 13A, B**). However, the HMOs has no effect on the NF-κB pathway in SNP MRSA-exposed macrophages (**Figure 5**). These results indicate that HMOs, have the potential to enhance the immune response against *S. aureus* (WT MRSA), without causing aberrant activation of naïve cells. To a lower extent, a similar effect is observed for *S. aureus* with SNPs related to antibiotic resistance (SNP MRSA).

## Discussion

The formation of the immune systems starts already in the embryonic phase within the uterus. However, the immune system of the newborn infant is still immature and relies heavily on external stimuli, e.g. stimuli from the microbiota, in order to fully develop (60). HMOs in breast milk have prebiotic functions and help shape and develop the gut eco system (61–63), and thereby the immune system, as the microbiota composition regulate specific immune responses. Vogel et al. showed that *S. aureus* enhanced a Th1 response, whereas *Bifidobacterium*, a classic infant-associated bacteria, had a mitigating effect on this immune response (64). HMOs are known to have a bifidogenic effect and can therefore indirectly support this microbiome-driven immune regulation. HMOs have also shown to affect immune cells, even without microbiota present, highlighting an effect that does not rely on microbial derivatives (15). Data have shown that HMOs have beneficial effects against various diseases such as rotavirus-induced diarrhea and necrotizing enterocolitis (65–67). It is also known that HMOs help shape the immune system in babies, as illustrated in a study by Goehring et al. where HMO-supplementation of infant formula decreased plasma levels of pro-inflammatory cytokines, bringing them closer to those of breastfed infants (20). Sialylated HMOs have been shown to increase the clearance of *Pseudomonas aeruginosa* in mice and in THP1 monocytes (68), and Xiao et al. demonstrated that a pool of HMOs have immunomodulatory functions on dendritic cells (18). HMOs have also been shown to be present in the amniotic fluid during pregnancy and could potentially already play a role in the development and modulation of the immune system in the embryonic state, and not only after birth (69).

While the effect of HMOs on the gut microbiota and their role in gut health is relatively well studied, the direct impact of HMOs on immune cells is still poorly understood. Here, we aimed to evaluate the direct effect of three individual HMOs with structures belonging to either the neutral, fucosylated or sialylated group. We found that HMOs can augment the response against *S. aureus*, illustrated by increased M1 surface markers, decreased expression of M2 markers (**Figure 3**), increased release of pro-inflammatory cytokines (**Figure 4**), enhanced NF-κB activation (**Figure 5**) and *S. aureus* phagocytosis (**Figure 7**; **Supplementary Figure 13**). We found that sialylated 6’SL had the highest immunomodulatory effect compared to the non-fucosylated neutral core HMO, LNnT and the fucosylated neutral core HMO, 2’FL. In parallel to our study, Boll et al. recently showed that M1 macrophages were strongly impacted by sialylated HMOs in response to LPS (19), through enhanced secretion of the inflammatory cytokines as IL-1β, IL-6 and TNF-α (19). This is similar to findings in our study, when challenging the cells with *S. aureus*, pointing to an immunomodulatory role for sialylated HMOs.

This study shows that HMOs can directly affect immune cells, though the molecular mode of action remains elusive. Hassan et al. demonstrated that the HMO 3’SL has several interactions with proteins found in murine macrophages, including well known glycan binding receptors as galectins (30). We hypothesize that HMOs work as surrogate TLR ligands that support a homeostatic immune activation, which is likely important for development of immune cells. In line with this, Kurakevich et al. observed that 3’SL mediated stimulatory effect on dendritic cells that was dependent on TLR4 (70). We did, however, not see changes in the surface expression of neither TLR2 nor TLR4 in this study. As previously mentioned, glycan-binding receptors, such as lectins and siglecs, are able to bind HMOs (28, 29, 32). In this study, a downregulation of CD169 (Siglec-1) was found, when macrophages were treated with HMOs and exposed to *S. aureus*, indicating a link between HMO-dependent macrophage activation and Siglec-1. We also found an increase in CD18 (**Figure 3C**), which is part of the important macrophage adhesion molecule Mac-1 (71). Surface expression ofCD11b (the other part of Mac-1) was however not changed (**Supplementary Figure 8A**). The HMO-mediated M1-like phenotype was highly linked to the presence of *S. aureus*. This indicates that the HMOs primarily work through co-stimulation, hypothetically through epigenetic regulation and dual-dependency between 6’SL and *S. aureus*, or through metabolic regulation of NF-κB, both interesting issues that needs further examination in further studies. Similarly, Ayechu-Muruzabal et al. demonstrated that 2′FL enhanced IFN-γ and IL-10 secretion in activated peripheral blood mononuclear cells co-cultured with the intestinal cell line HT-29, but only in the presence of CpG (mimicking bacterial trigger), whereas cytokine secretion was not affected by exposure to 2’FL alone (72).

In our study, we saw a minor change in the proliferation profile of macrophages by 6’SL, while this was consistently observed, we have to stress that the effect was small. The cell cycle phase is indeed important for macrophage plasticity and polarization (73). Further, IL-4 can lead to proliferation of macrophages (74), however this is likely not the causative effect of 6’SL, as the all HMOs examined in the current study induced similar levels of IL-4.

Human milk possesses many relevant immunomodulators including HMOs and antibodies (1). Current investigations also indicate that milk contains antigen fragments, believed to help develop and train the immune response in infants (75, 76). Previously, it was believed that the memory of the immune system was confined to the adaptive immune cells, and only developed as a response to infectious encounters. However, recent years, trained immunity of the innate immune cells has been highly investigated, challenging the assumption that memory is solely limited to cells of the adaptive immune system. It reveals that innate cells exposed to, and activated by, certain stimuli can enhance their response when encountering a non-specific pathogen at a later stage through epigenetic changes in the cell (77). Trained immunity can lead to enhanced effector functions such as improved phagocytosis and increased cytokine release upon pathogen encounter. Such booster effects of the infant immune system could be highly relevant in combatting infectious diseases. It has been shown that β-glucans can train the innate immune cells, such as monocytes and macrophages, to enhance the immune response at a later state (78, 79). Like β-glucans, HMOs possess the ability to bind pattern recognition receptors (28, 29). This, together with the immune-enhancing effects seen in this study, suggests that HMOs could have a role in developing and training innate cells of young infants, improving their ability to overcome infections. The impact of HMOs on trained immunity should be considered and investigated further.

Monocytes and macrophages are essential in pathogen clearing and are especially important in bacterial infections including clearance of *S. aureus* (44). Persistent activation of M2 macrophages is associated with persistent infections as well as biofilm formation (80). *S. aureus* has developed multiple mechanisms to manipulate and evade the host immune system (43), e.g.; *S. aureus* derived lactate-production can reprogram the immune response and maintain persistent infection (81). Therefore, it is highly relevant to identify methods to enhance M1-like effects against *S. aureus*. One way to augment the M1-response is by the use of metabolic reprogramming, as illustrated by Yamada et al. (47). In the current study, the immune-enhancing effect of HMOs were similar but lower for SNP MRSA compared to WT MRSA, indicating that the HMOs will have the same effect on different MRSA strains. As antibiotic-resistance and immune evasion are an increasing problem for *S. aureus*, alternatives to antibiotic treatments that influence activation of the immune response are of great interest and should be investigated further. Since HMOs have a well-regarded safety profile and are well-tolerated, this study supports a possible continuous use of HMOs to limit *S. aureus* infections, in infants, but also in elderly or people with immune defects.

In summary, this study characterized specific effects of a neutral, fucosylated and sialylated HMO structure on human monocyte derived macrophages in response to *S. aureus*, and we saw that HMOs have the potential to enhance the myeloid immune response against bacteria. Importantly, even though all HMOs were able to activate macrophages, the different HMO structures showed various degree of myeloid activation, which underlines the diversity of the different HMOs. This is further exemplified by a recent study by Boll et al. demonstrating particular biological properties depending on HMO structural class (19). Taken together, our study suggests that HMOs are able to enhance the immune response under infectious circumstances, without causing notable activation of naïve cells, highlighting a great potential of HMOs as a non-antibiotic alternative against infectious diseases.

Further studies are needed to assess the influence of HMOs on other immune cell populations. For example, HMOs were shown to dose-dependently reduce Mac-1 in neutrophils (82), which can have important implications for neutrophil-mediated pathogen clearance such as *S. aureus*-induced pneumonia (83). Moreover, the context of HMO exposure of macrophages and their precursors can have consequences for their clearance of infectious agents (84, 85).

## Data availability statement

The original contributions presented in the study are included in the article/**Supplementary Material**, further inquiries can be directed to the corresponding author.

## Ethics statement

Ethical approval was not required for the studies involving humans because Blood was purchased from National Hospital blood bank. The studies were conducted in accordance with the local legislation and institutional requirements. Written informed consent to participate in this study was not required from the participants in accordance with the national legislation and the institutional requirements.

## Author contributions

SJ: Conceptualization, Data curation, Formal analysis, Funding acquisition, Investigation, Methodology, Project administration, Resources, Visualization, Writing – original draft, Writing – review & editing. AL: Data curation, Formal analysis, Investigation, Methodology, Project administration, Visualization, Writing – original draft, Writing – review & editing. MM: Formal analysis, Investigation, Methodology, Resources, Validation, Writing – original draft, Writing – review & editing. LA: Conceptualization, Formal analysis, Funding acquisition, Investigation, Methodology, Writing – original draft, Writing – review & editing. KC: Conceptualization, Funding acquisition, Investigation, Methodology, Project administration, Resources, Supervision, Writing – original draft, Writing – review & editing. SS: Conceptualization, Funding acquisition, Investigation, Methodology, Project administration, Resources, Supervision, Writing – original draft, Writing – review & editing.
