# Supplementary material for: Human milk oligosaccharides regulate human macrophage polarization and activation in response to Staphylococcus aureus
Source: Front Immunol. 2024 Jun 6;15:1379042. doi: 10.3389/fimmu.2024.1379042 (PMC11187579; doi:10.3389/fimmu.2024.1379042)
Supplement: Supplementary file 1 [file DataSheet_1.pdf]

Supplementary Material

Supplementary table 1: Antibodies used for flow cytometry.

**Marker**	**Catalogue number**	**Isotype**	**Provider**	**Color**
TLR2	130-127-882	Rea IgG1	Miltenyi Biotec	PE
CD206	311110	IgG1	Biolegend	APC
CD200R	12-9201-42	IgG1	eBioscience	PE
SIRPa	130-123-742	Rea IgG1	Miltenyi Biotec	PE
CD14	130-110-518	Rea IgG1	Miltenyi Biotec	FITC
CD169	130-121-111	Rea IgG1	Miltenyi Biotec	APC-Vio 770
CD163	130-112-134	Rea IgG1	Miltenyi Biotec	Vioblue
CD86	130-116-166	Rea IgG1	Miltenyi Biotec	Vioblue
CD209	551545	IgG2b	BD Biosciences	APC
CD11b	130-110-556	Rea IgG1	Miltenyi Biotec	APC-vio 770
CD80	130-117-719	Rea IgG1	Miltenyi Biotec	APC
CD36	130-110-741	Rea IgG1	Miltenyi Biotec	APC
CD18	130-119-091	Rea IgG1	Miltenyi Biotec	Pe-vio-770
TLR4	12-9917-42	IgG2a	eBioscience	PE
CD16	130-113-393	Rea IgG1	Miltenyi Biotec	PE
PDL1	17-5983-42	IgG1	eBioscience	APC
CD137L	130-212-337	IgG1	Miltenyi Biotec	APC

Supplementary table 2: Isotype controls used for flow cytometry.

**Marker**	**Catalogue number**	**Isotype**	**Provider**	**Fluorescence**
Isotype	130-113-454	IgG1 rea	Miltenyi Biotec	Vioblue
Isotype	130-113-452	IgG1 rea	Miltenyi Biotec	Pe vio 770
Isotype	130-113-446	IgG1 rea	Miltenyi Biotec	APC
Isotype	130-113-450	IgG1 rea	Miltenyi Biotec	PE
Isotype	130-113-449	IgG1 rea	Miltenyi Biotec	FITC
Isotype	130-130-447	IgG1 rea	Miltenyi Biotec	APC vio 770
Isotype	555751	IgG1	BD Biosciences	APC
Isotype	555745	IgG2b	BD Biosciences	APC
Isotype	555749	IgG1	BD Biosciences	PE
Isotype	555574	IgG2a	BD Biosciences	PE

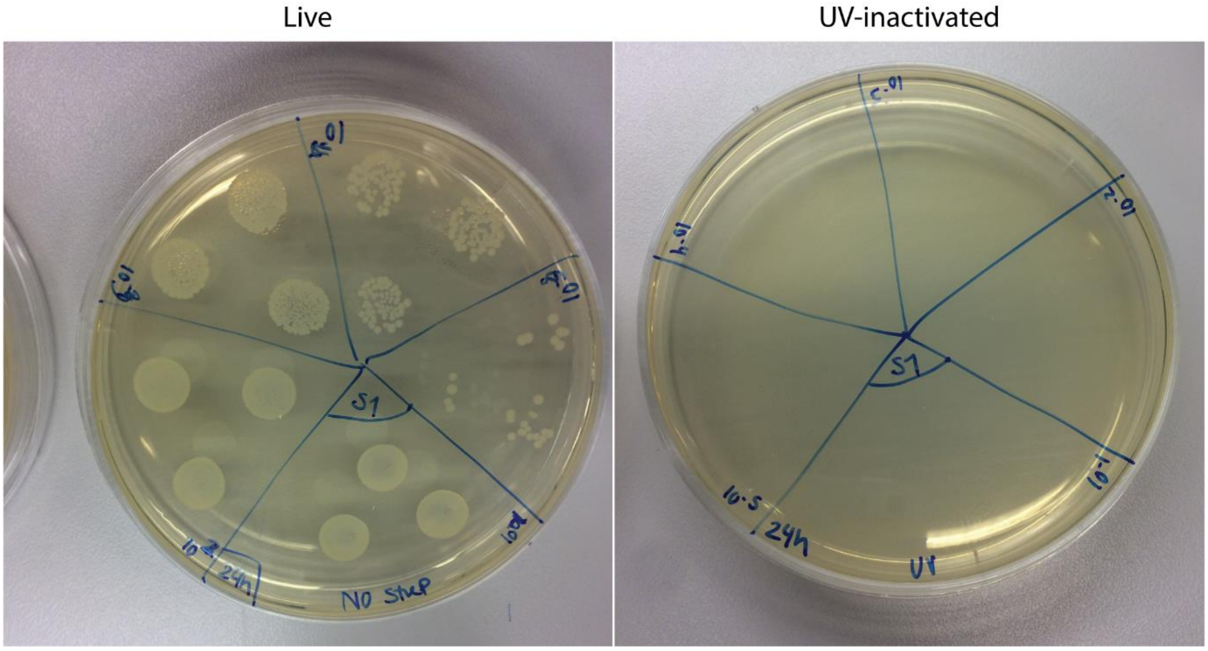

Supplementary figure 1: Verification of UV-inactivation of *S. aureus.* *S. aureus* colonies were grown to early stationary phase, set up in PBS to OD600 = 1 and subjected to pulsed UV for inactivation. Bacterial death was verified by plating and incubating on TSA plates at 37 °C overnight.

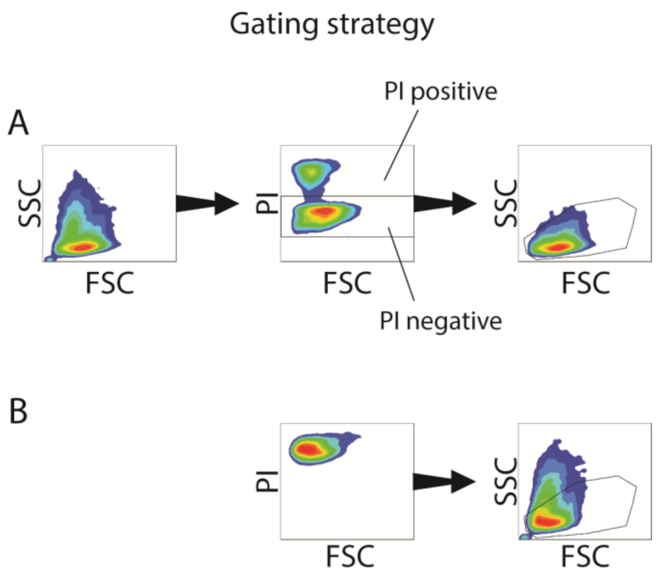

Supplementary figure 2: Gating strategy. Flow cytometry data was analyzed in one of the following ways: A) Gating on PI-negative (viable) cells or B) gating in FSC-SSC plot from the gate made on PI negative cells. Gating strategy B was only used when the PI staining was unusable due to technical issues or overflow from other fluorescent channels. FSC: Forward Scatter ; SSC: Side Scatter ; PI: Propidium iodide

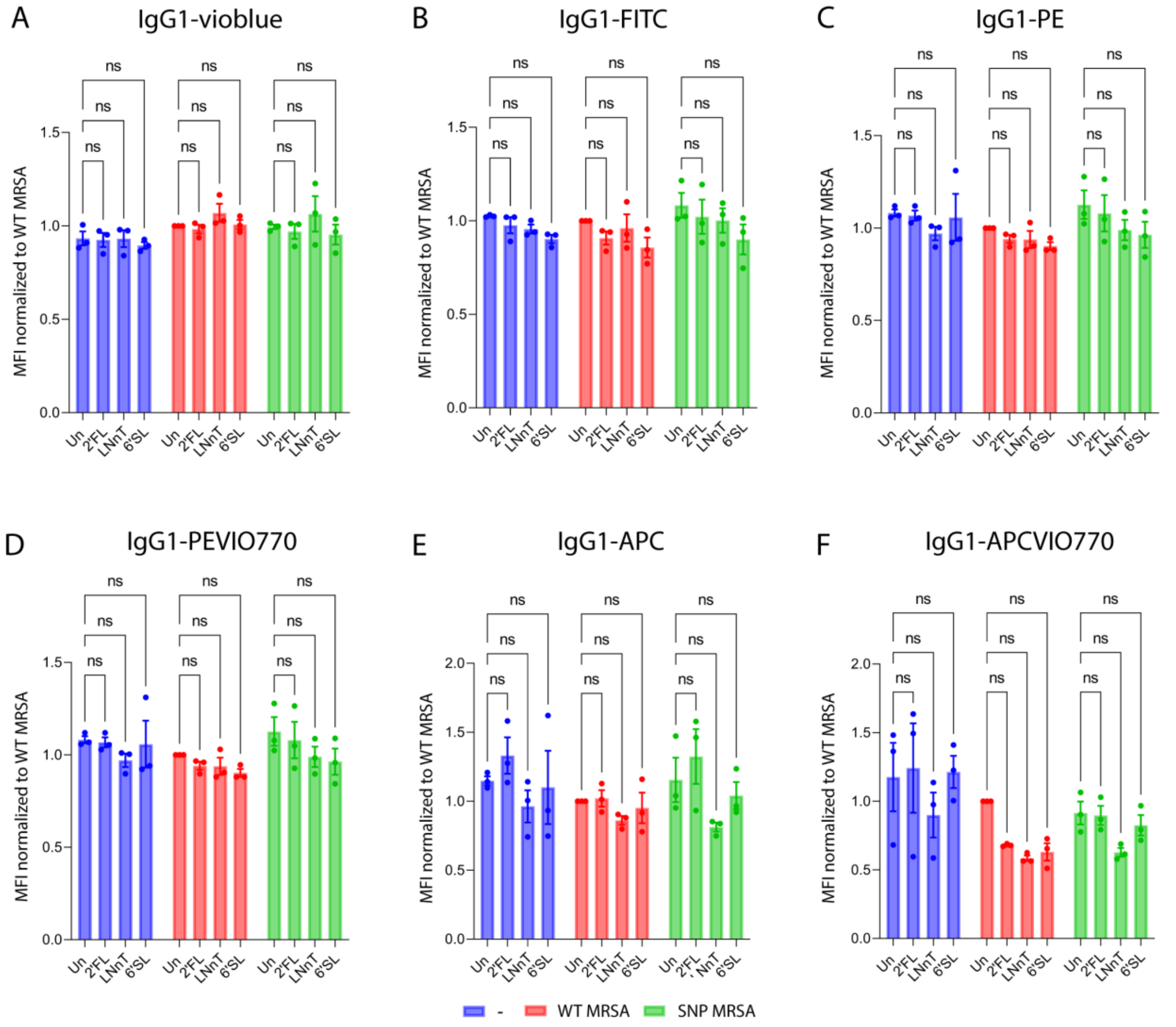

Supplementary figure 3: Isotype controls. Monocytes were treated with HMOs followed by treatment with WT MRSA and SNP MRSA and analyzed by flow cytometry on day 8. Data is presented as bar graphs with data points showing cell surface expression of isotype controls A) IgG1-Vioblue, B) IgG1-FITC, C) IgG1-PE, D) IgG1-PE-Vio770, E) IgG1-APC and F) IgG1-APC_Vio770 from three independent experiments. Data is presented as MFI values ± SEM, normalized to WT MRSA untreated samples. Isotype controls were included in all flow cytometry studies. Statistical analysis was performed by 2-way ANOVA with Dunnett's multiple comparison test and presented relative to untreated control. MFI: Mean Fluorescent Intensity ; ns: not significant ; Un: Untreated ; Un: untreated ; 2'FL: 2'-Fucosyllactose ; LNnT: Lacto-N-neotetraose ; 6'SL: 6'-Sialyllactose.

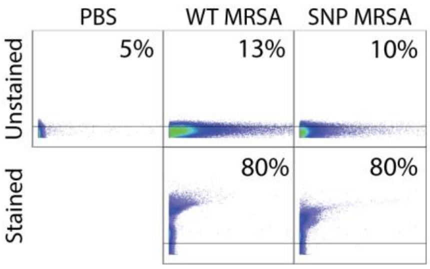

Supplementary figure 4: SE-AF647-labelled WT MRSA and SNP MRSA.
UV inactivated *S. aureus* strains were labelled with SE-AF647. The staining was confirmed by flowcytometry. Data is depicted as dot plots showing the negative PBS control, the unstained controls and the stained *S. aureus* strains and confirms staining of the WT MRSA and SNP MRSA.

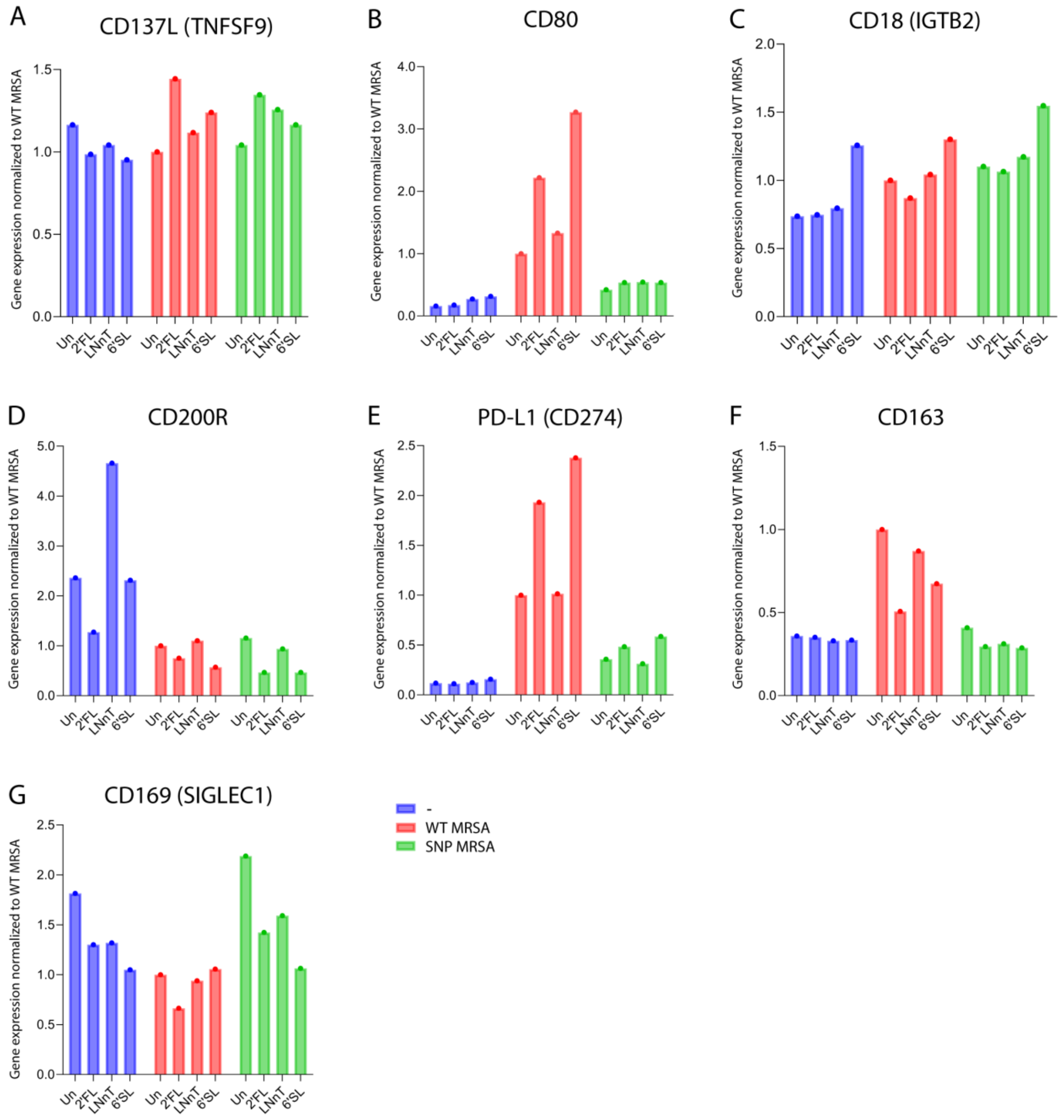

Supplementary figure 5: HMOs alter gene expression of several macrophage relevant proteins in response to *S. aureus*. Monocytes were treated with HMOs followed by treatment with WT MRSA or SNP MRSA. On day 8, total RNA was isolated, followed by transcriptomic profiling. Data is presented as bar graphs with data points showing gene expression of A) TNFSF9, B) CD80, C) ITGB2 D) CD200R, E) CD274, F) CD163 and G) SIGLEC1 from one experiment. Data is presented as gene-expression normalized to WT MRSA Un. Un: Untreated ; 2’FL: 2’-Fucosyllactose ; LNnT: Lacto-N-neotetraose ; 6’SL: 6’-Sialyllactose.

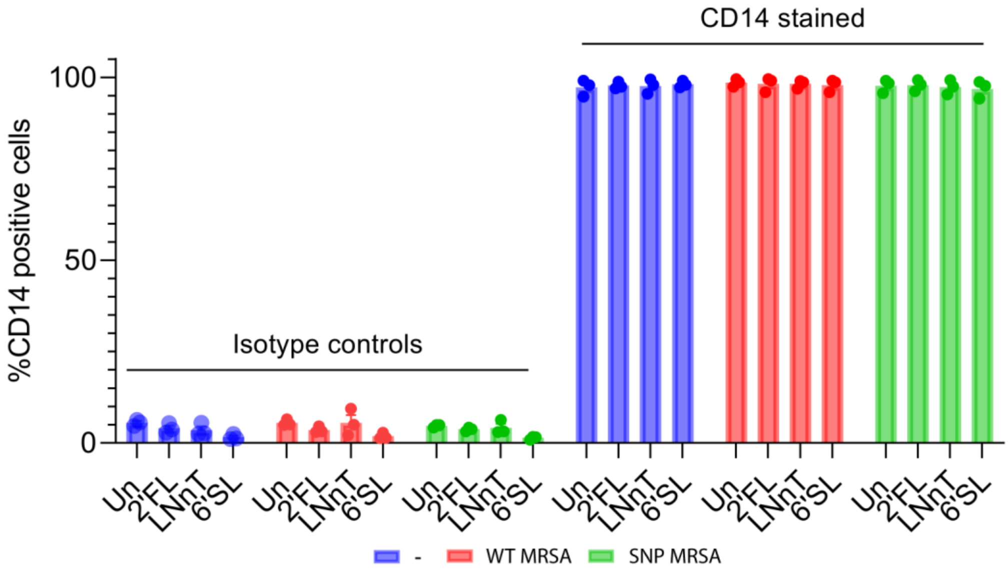

Supplementary figure 6: All monocyte-derived macrophages are CD14 positive. Monocytes were treated with HMOs followed by treatment with WT MRSA and SNP MRSA and analyzed by flow cytometry on day 8. Data is presented as a bar graph with data points showing cell surface expression of CD14 from three independent experiments. Data is presented as %positive CD14 cells, set to 5% to specific isotype control ± SEM. Un: Untreated; 2'FL: 2'-Fucosyllactose ; LNnT: Lacto-N-neotetraose ; 6'SL: 6'-Sialyllactose.

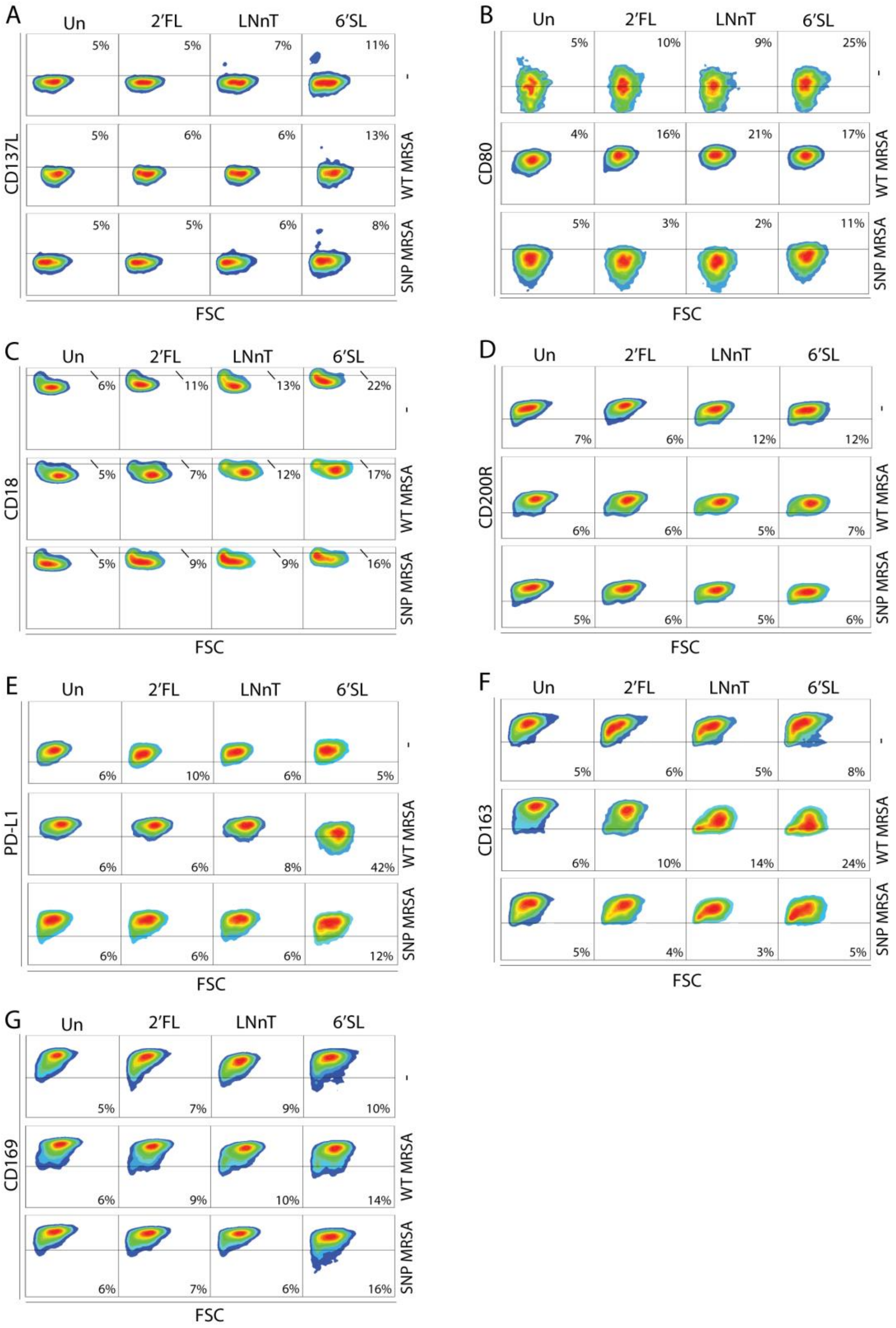

Supplementary Figure 7: Treatment with HMOs affects surface expression and polarization of macrophages. Monocytes were treated with HMOs followed by treatment with WT MRSA and SNP MRSA and analyzed by flow cytometry on day 8. Data is presented as dot plots showing cell surface expression of A) CD137L, B) CD80, C) CD18, D) CD200R, E) PD-L1, F) CD163 and G) CD169. Data is shown from one experiment but is representative for A) two independent experiments and B-G) three independent experiments. The grid is set to ~5% for the untreated sample. The grid is set above or below the cell population according to measurements of either up- or downregulation, respectively. Un: Untreated ; 2'FL: 2'-Fucosyllactose ; LNnT: Lacto-N-neotetraose ; 6'SL: 6'-Sialyllactose ; FSC: Forward Scatter ; SSC: Side Scatter.

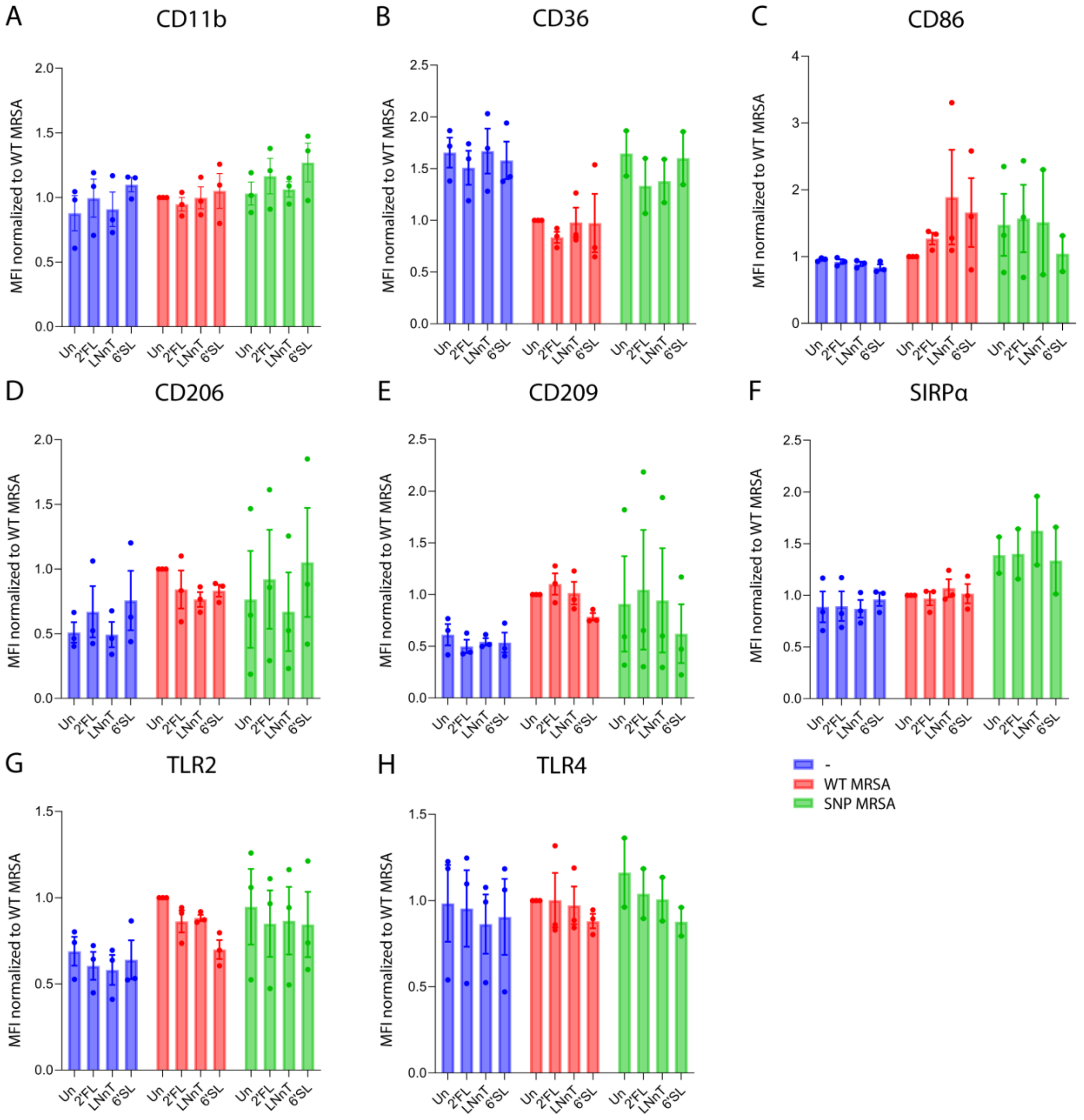

Supplementary figure 8: Classical macrophage surface markers show no difference related to HMOs. Monocytes were treated with HMOs followed by treatment with WT MRSA and SNP MRSA and analyzed by flow cytometry on day 8. Data is presented as bar graphs with data points showing cell surface expression of A) CD11b, B) CD36, C) CD86, D) CD206, E) CD209, F) SIRPα G) TLR2 and H) TLR4 from three independent experiments. Data is presented as MFI values ± SEM, normalized to WT MRSA untreated samples. Statistical analysis was performed by 2-way ANOVA with Dunnett's multiple comparison test and presented relative to untreated control but showed no significant differences. MFI: Mean Fluorescent Intensity ; Un: Untreated ; 2'FL: 2'-Fucosyllactose ; LNnT: Lacto-N-neotetraose ; 6'SL: 6'-Sialyllactose.

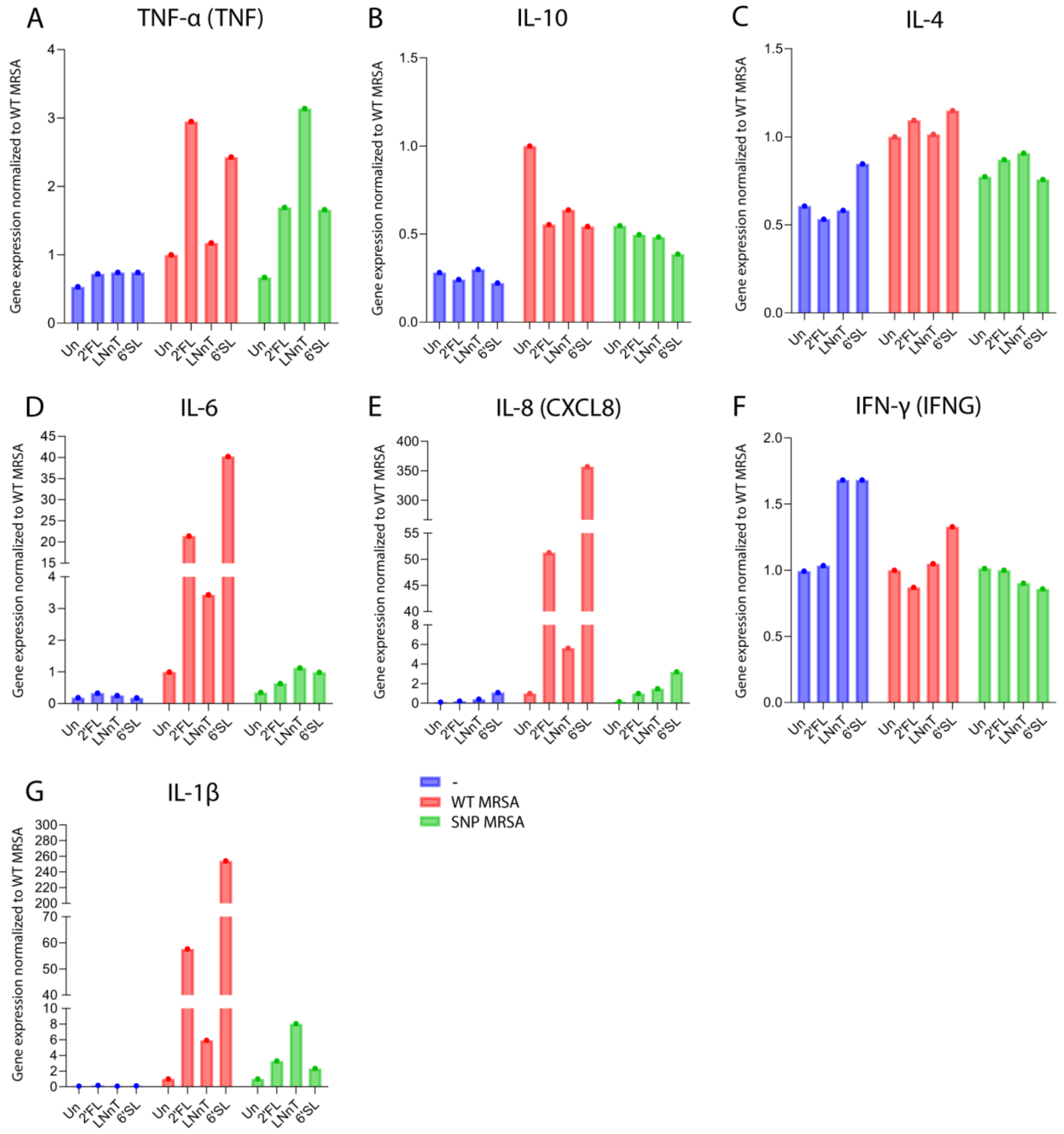

Supplementary figure 9: HMOs alter gene expression of several classical macrophage cytokines in response to *S. aureus*. Monocytes were treated with HMOs followed by treatment with WT MRSA and SNP MRSA. On day 8, total RNA was isolated, followed by transcriptomic profiling. Data is presented as bar graphs with data points showing gene expression of A) TNF, B) IL-10, C) IL-4 D) IL-6, E) IL-8, F) IFN-γ and G) IL-1β from one experiment. Data is presented as gene-expression normalized to WT MRSA Un. Un: Untreated ; 2'FL: 2'-Fucosyllactose ; LNnT: Lacto-N-neotetraose ; 6'SL: 6'-Sialyllactose.

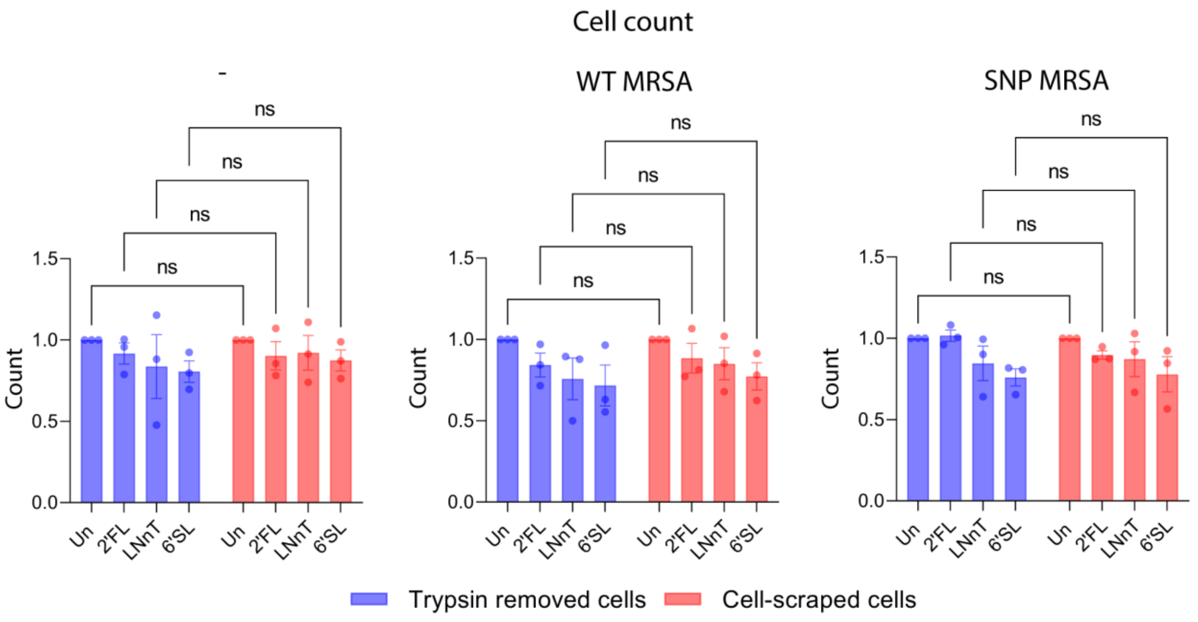

Supplementary figure 10: There is no difference between citric saline treatment combined with cell-scraping and trypsination of macrophages in relation to cell number.

Monocytes were treated with HMOs followed by treatment with WT MRSA and SNP MRSA and counted by flow cytometry. Data is presented as bar graphs with data points showing cell number from three independent experiments. Data is presented as counts ± SEM, normalized to WT MRSA untreated samples. Statistical analysis was performed by 2-way ANOVA with Sidak's multiple comparison test and showed no difference in cell number between trypsination and cell scraping. Un: Untreated; 2'FL: 2'-Fucosyllactose ; LNnT: Lacto-N-neotetraose ; 6'SL: 6'-Sialyllactose ; ns: not significant.

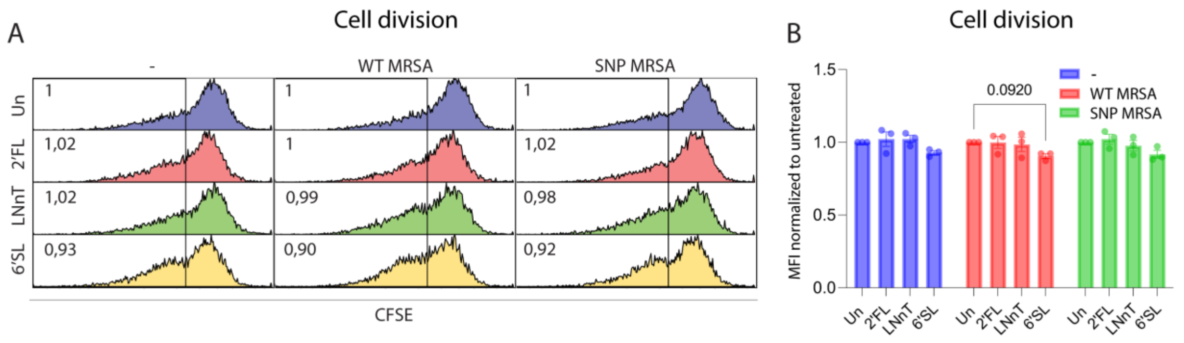

Supplementary figure 11: Treatment with HMOs changes the proliferation of primary macrophages. Monocytes were stained with CFSE and treated with HMOs followed by treatment with WT MRSA and SNP MRSA. On day 8, cell proliferation was measured by flow cytometry. The figure shows A) Histograms displaying proliferation from one donor representative of three independent studies. The numbers indicate the collected relative change in MFI compared to untreated sample from three independent experiments. B) Bar graph with data points ± SEM showing CFSE-MFI normalized to untreated sample from three independent experiments. Statistical analysis was performed by 2-way ANOVA with Dunnett's multiple comparison test showing no significance. CFSE: Carboxyfluorescein succinimidyl ester ; MFI: Mean Fluorescent Intensity ; Un: Untreated ; 2'FL: 2'-Fucosyllactose ; LNnT: Lacto-N-neotetraose ; 6'SL: 6'-Sialyllactose.

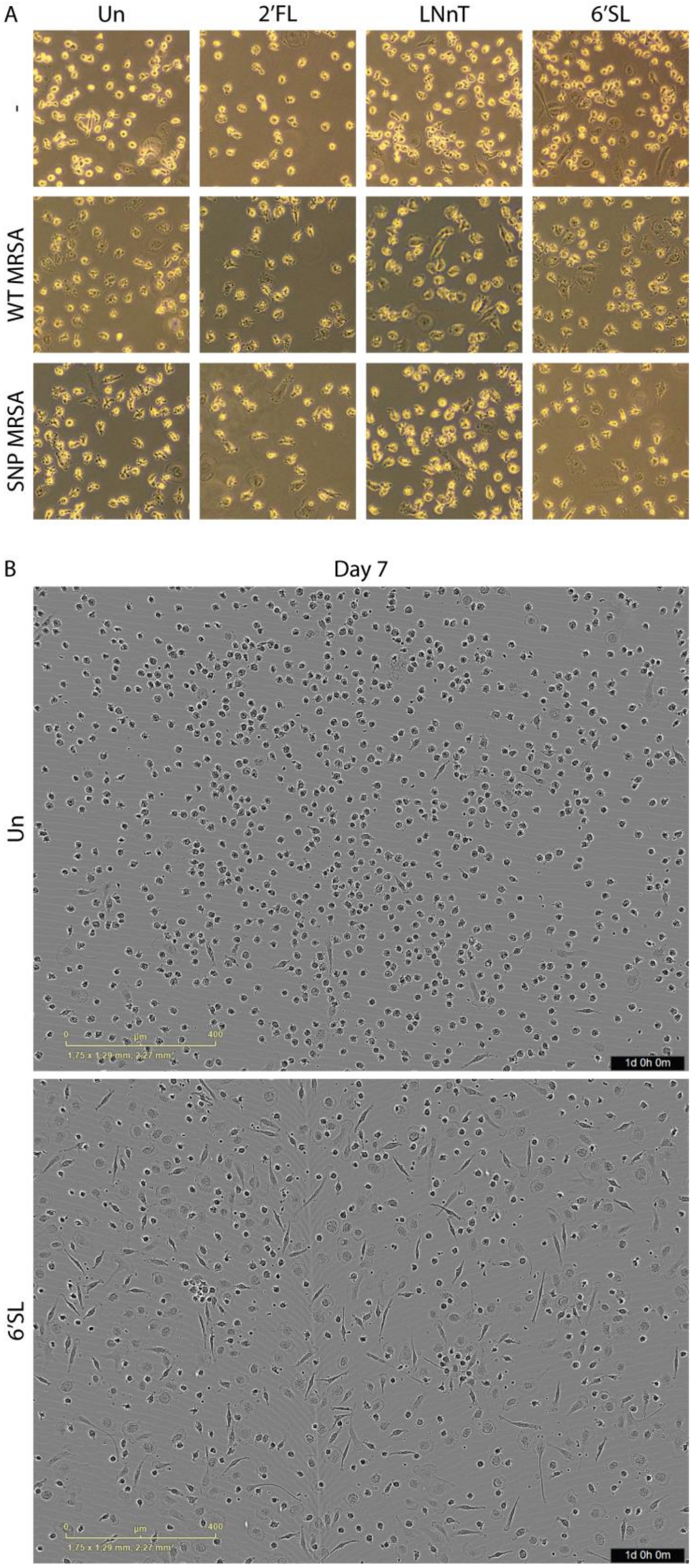

Supplementary figure 12: HMO treatment alters morphology of monocyte-derived macrophages. Monocytes were treated with HMOs followed by treatment with WT MRSA and SNP MRSA and A) photographed in the microscope on day 8 or B) in the Incucyte on day 7. Data is presented as photos of monocyte-derived macrophages treated with HMOs. The data presents photos from one donor but represents findings from A) eight and B) four individual donors. Un: Untreated; 2'FL: 2'-Fucosyllactose ; LNnT: Lacto-N-neotetraose ; 6'SL: 6'-Sialyllactose.

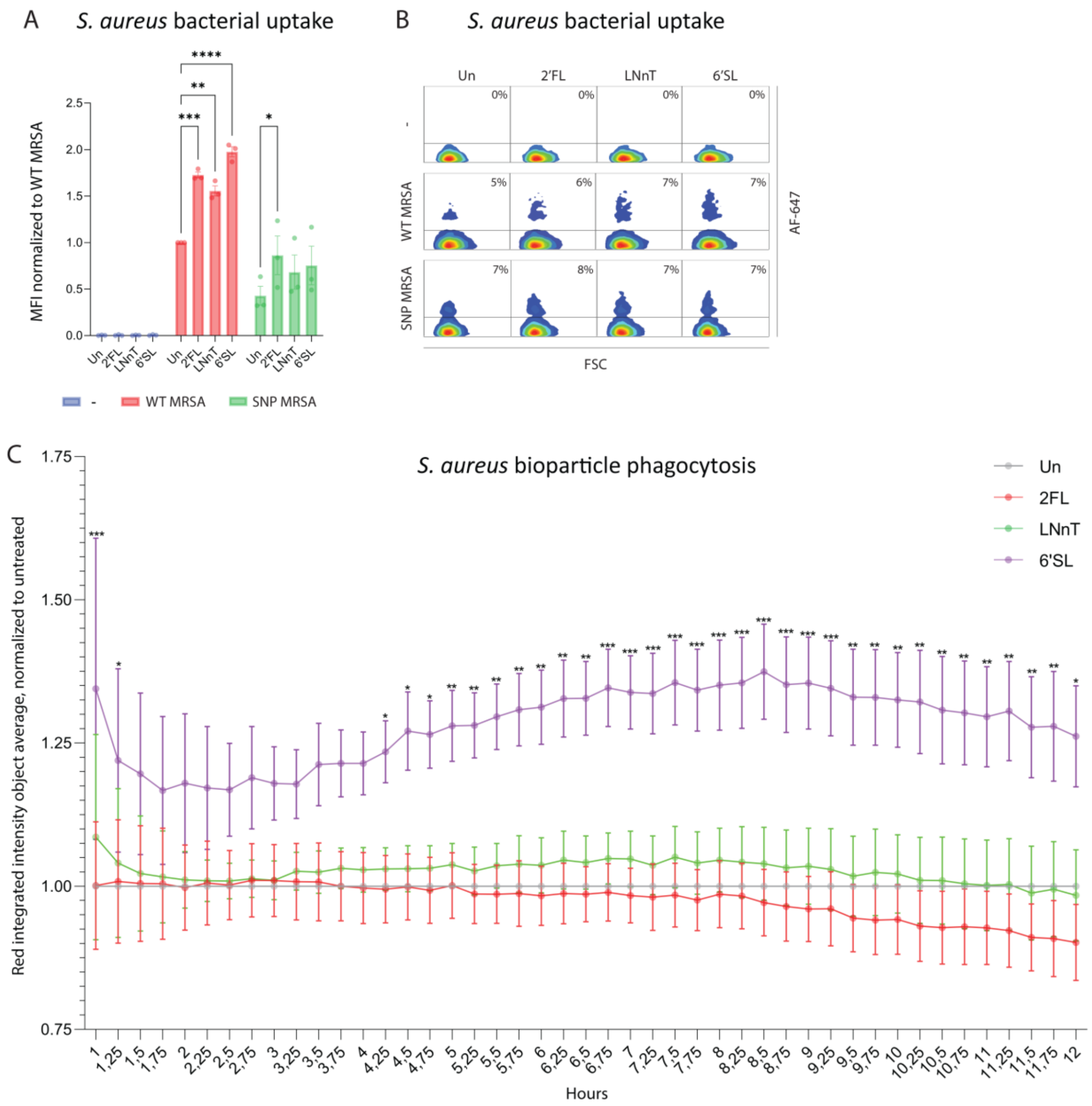

Supplementary figure 13: Treatment with HMOs increases uptake of S. aureus in U937 monocytic cell line and phagocytosis of S. aureus bioparticles in primary macrophages. A+B) The U937 cell line was treated with HMOs followed by treatment with fluorescent-labelled WT MRSA and SNP MRSA and analyzed by flow cytometry. Data is presented as A) bar graph with data points showing uptake of fluorescent-labelled *S. aureus* ± SEM from three individual experiments and B) dot plots of uptake from one experiment, representative for three experiments. Statistical analysis was performed by 2-way ANOVA with Dunnett's multiple comparison test and presented relative to WT MRSA untreated sample. C) Primary macrophages were cultured for 8 days in the presence of 20 mM HMOs and 40 ng/mL M-CSF. On day 8 *S. aureus* bioparticles were added to the cells and the uptake was analyzed for 12 hours. Data is presented as a line graph representing the red integrated intensity object average over time for four donors ± SEM. Statistical analysis was performed by 2-way ANOVA with Dunnett's multiple comparison test and presented relative to untreated sample. Data used in C) is the continuous measurements of the data depicted in the bar graph in figure 7A. MFI: Mean Fluorescent Intensity ; Un: Untreated ; 2'FL: 2'-Fucosyllactose ; LNnT: Lacto-N-neotetraose ; 6'SL: 6'-Sialyllactose. *$p < 0.05$, **$p < 0.01$, ***$p < 0.001$.
